# Supplementary material for: Study protocol: the ear–nose–throat (ENT) prospective international cohort of patients with primary ciliary dyskinesia (EPIC-PCD)
Source: BMJ Open. 2021 Oct 11;11(10):e051433. doi: 10.1136/bmjopen-2021-051433 (PMC8506890; doi:10.1136/bmjopen-2021-051433)
Supplement: Supplementary data [file bmjopen-2021-051433supp001.pdf]

Supplementary file: Ethics

The study has been reviewed and approved by the local Human Research Ethics Committees at every participating centre, based on local legislation. We list below the names of the ethics committees which approved the study and the approval reference numbers, when applicable.

A. The following centres have a pre-existing or new **ethical approval, which allows the contribution of pseudonymised data to observational collaborative international studies (covers the EPIC-PCD study)**:

- University Children´s Hospital Charité-Universitätsmedizin, **Berlin, Germany:** Ethical Committee Charité (EA2/003/21)
- University Children's hospital, **Bern, Switzerland**: Cantonal Ethics Committee of Bern (KEK-BE: 060/2015)
- University of **Cyprus:** Ethical Committee for biomedical research in Leukosia Cyprus (ΕΕΒΚ/ΕΠ/2013/21)
- Marmara University **Istanbul, Turkey:** Ethical Committee of Marmara University (09.2018.395)
- University Hospital of **Southampton, United Kingdom:** Southampton and South West Hampshire research ethics committee (06/Q1702/109)

B. The following centres applied for **ethical approval to participate specifically to the EPIC-PCD study**:

- VU University medical center (VUmc), **Amsterdam, The Netherlands:** The Medical Ethics Review Committee of VU University Medical Center reviewed the application and concluded on 24th of November 2020 that no approval is needed to participate to the EPIC-PCD cohort as the Medical Research Involving Human Subjects Act does not apply to the study.
- Hacettepe University, **Ankara, Turkey:** Non-interventional clinical research EC of Hacettepe University (2020/11-47)
- University Hospital of **Leuven, Belgium**: Ethical Committee for Research of University Hospitals Leuven (S64411)
- Hospital Universitario La Fe in **Valencia, Spain:** Ethical Committee of medical investigations of Hospital Universitario La Fe (2020-498-1)
- University Hospital Bicetre Paris-Sud and Centre Hospitalier Intercommuncal Creteil**, Paris, France:** Both hospitals belong to the Assistance Publique–Hôpitaux de Paris and applied for joint approval. The AP-HP Direction de la Recherche Clinique et de l'Innovation reviewed the application and concluded on 4th of February 2021 that that no approval is needed to participate to the EPIC-PCD cohort as the Jardé law that regulates clinical research in France does not apply to the study.
