# Supplementary material for: Study protocol: the ear–nose–throat (ENT) prospective international cohort of patients with primary ciliary dyskinesia (EPIC-PCD)
Source: BMJ Open. 2021 Oct 11;11(10):e051433. doi: 10.1136/bmjopen-2021-051433 (PMC8506890; doi:10.1136/bmjopen-2021-051433)
Supplement: Author's manuscript [file bmjopen-2021-051433.draft_revisions.pdf]

BMJ Open is committed to open peer review. As part of this commitment we make the peer review history of every article we publish publicly available.

When an article is published we post the peer reviewers' comments and the authors' responses online. We also post the versions of the paper that were used during peer review. These are the versions that the peer review comments apply to.

The versions of the paper that follow are the versions that were submitted during the peer review process. They are not the versions of record or the final published versions. They should not be cited or distributed as the published version of this manuscript.

BMJ Open is an open access journal and the full, final, typeset and author-corrected version of record of the manuscript is available on our site with no access controls, subscription charges or pay-per-view fees (http://bmjopen.bmj.com).

If you have any questions on BMJ Open's open peer review process please email info.bmjopen@bmj.com

BMJ Open

Study protocol: the Ear-Nose-Throat (ENT) Prospective International Cohort of patients with Primary Ciliary dyskinesia (EPIC-PCD)

Journal:	*BMJ Open*
Manuscript ID	bmjopen-2021-051433
Article Type:	Protocol
Date Submitted by the Author:	22-Mar-2021

Complete List of Authors:	Goutaki, Myrofora ; Universitat Bern, Institute of Social and Preventive Medicine; Universitat Bern, Paediatric Respiratory Medicine, Children's University Hospital of Bern<br>Lam, Yin Ting; Institute of Social and Preventive Medicine, University of Bern<br>Alexandru, Mihaela; AP-HP, Hôpital Kremlin-Bicetre, Service d'ORL et de Chirurgie Cervico-Faciale; Faculté de Médecine, Université Paris-Saclay<br>Anagiotos, Andreas; Department of Otorhinolaryngology, Nicosia General Hospital<br>Armengot, Miguel; Department of Otorhinolaryngology, and Primary Ciliary Dyskinesia Unit, La Fe University and Polytechnic Hospital; Medical School, Valencia University<br>Bequignon, Emilie; AP-HP, Hôpital Henri Mondor et Centre Hospitalier Intercommunal de Créteil, Service d'Oto-Rhino-Laryngologie et de Chirurgie Cervico-Faciale<br>Boon, Mieke; Department of Paediatrics, University Hospital<br>Burgess, Andrea; Primary Ciliary Dyskinesia Centre, Southampton Children's Hospital, Southampton NHS Foundation Trust<br>Coste, Andre; AP-HP, Hôpital Henri Mondor et Centre Hospitalier Intercommunal de Créteil, Service d'Oto-Rhino-Laryngologie et de Chirurgie Cervico-Faciale; Faculté de Médecine, Université Paris-Est Créteil, Institut National de la Santé et de la Recherche Médicale, Centre National de la Recherche Scientifique, Hôpital Henri Mondor<br>Emiralioglu, Nagehan; Department of Pediatric Pulmonology, Hacettepe University, School of Medicine<br>Erdem, Ela; Department of Pediatric Pulmonology, Marmara University, School of Medicine<br>Lorent, Natalie; Department of Respiratory Diseases, University Hospital<br>Haarman, Eric G.; Department of pediatric pulmonology, Emma Children's Hospital, Amsterdam UMC, Vrije Universiteit<br>Harris, Amanda; Primary Ciliary Dyskinesia Centre, NIHR Respiratory Biomedical Research Centre, University of Southampton<br>Hool, Sara-Lynn; Department of Otorhinolaryngology, Head and Neck Surgery, University Hospital of Bern, University of Bern<br>Karadag, Bulent; Department of Pediatric Pulmonology, Marmara University, School of Medicine<br>Kim, Sookyung; AP-HP, Hôpital Kremlin-Bicetre, Service d'ORL et de Chirurgie Cervico-Faciale<br>Latzin, Philipp; Paediatric Respiratory Medicine, Children's University

	Hospital of Bern, University of Bern<br>Ozcelik, Ugur; Department of Pediatric Pulmonology, Hacettepe University, School of Medicine<br>Reula, Ana; Biomedical Sciences Department, CEU-Cardenal Herrera University; Molecular, Cellular and Genomic Biomedicine Group, IIS La Fe<br>Roehmel, Jobst; Department of Pediatric Pulmonology, Immunology and Critical Care Medicine, Charité-Universitätsmedizin<br>van Gogh, Christine; Department of Otolaryngology/ Head and Neck Surgery, Amsterdam University Medical Center<br>Yiallouros, Panayiotis; Medical School, University of Cyprus; Pediatric Pulmonology Unit, Hospital 'Archbishop Makarios III'<br>Zappe, Soeren; Department of Otorhinolaryngology, Head and Neck Surgery, Charité-Universitätsmedizin<br>PAPON, Jean; AP-HP, Hôpital Kremlin-Bicetre, Service d'ORL et de Chirurgie Cervico-Faciale; Faculté de Médecine, Université Paris-Saclay

Keywords:	Chronic airways disease < THORACIC MEDICINE, Epidemiology < THORACIC MEDICINE, RESPIRATORY MEDICINE (see Thoracic Medicine), Audiology < OTOLARYNGOLOGY, Paediatric otolaryngology < OTOLARYNGOLOGY, Paediatric thoracic medicine < THORACIC MEDICINE

SCHOLARONE™
Manuscripts

I, the Submitting Author has the right to grant and does grant on behalf of all authors of the Work (as defined in the below author licence), an exclusive licence and/or a non-exclusive licence for contributions from authors who are: i) UK Crown employees; ii) where BMJ has agreed a CC-BY licence shall apply, and/or iii) in accordance with the terms applicable for US Federal Government officers or employees acting as part of their official duties; on a worldwide, perpetual, irrevocable, royalty-free basis to BMJ Publishing Group Ltd ("BMJ") its licensees and where the relevant Journal is co-owned by BMJ to the co-owners of the Journal, to publish the Work in this journal and any other BMJ products and to exploit all rights, as set out in our licence.

The Submitting Author accepts and understands that any supply made under these terms is made by BMJ to the Submitting Author unless you are acting as an employee on behalf of your employer or a postgraduate student of an affiliated institution which is paying any applicable article publishing charge ("APC") for Open Access articles. Where the Submitting Author wishes to make the Work available on an Open Access basis (and intends to pay the relevant APC), the terms of reuse of such Open Access shall be governed by a Creative Commons licence – details of these licences and which Creative Commons licence will apply to this Work are set out in our licence referred to above.

Other than as permitted in any relevant BMJ Author's Self Archiving Policies, I confirm this Work has not been accepted for publication elsewhere, is not being considered for publication elsewhere and does not duplicate material already published. I confirm all authors consent to publication of this Work and authorise the granting of this licence.

For peer review only

Study protocol: the Ear-Nose-Throat (ENT) Prospective International Cohort of patients with Primary Ciliary Dyskinesia (EPIC-PCD)

Target Journal: BMJ Open
Second choice: ERJ Open Res

Manuscript: 3229/4000 words
References: 38
Display items: 2 figures, 1 text box

Suggested reviewers:

Adam Shapiro, Margaret Leigh (North American PCD Network)

Suzanne Crowley, Celine Kempeneers (European PCD network)

Basile Landis, Isao Suzaki (ENT specialists not PCD experts but might find it interesting)

For peer review only

For peer review only - http://bmjopen.bmj.com/site/about/guidelines.xhtml

Title page

Study protocol: the Ear-Nose-Throat (ENT) Prospective International Cohort of patients with Primary Ciliary Dyskinesia (EPIC-PCD)

Myrofora Goutaki[1-2], Yin Ting Lam[1], Mihaela Alexandru[3-4], Andreas Anagiotos[5], Miguel Armengot[6-7], Emilie Bequignon[8], Mieke Boon[9], Andrea Burgess[10], Andre Coste[8,11], Nagehan Emiralioglu[12], Ela Erdem[13], Eric G. Haarman[14], Amanda Harris[15], Sara-Lynn Hool[16], Bulent Karadag[13], Sookyung Kim[3], Philipp Latzin[2], Natalie Lorent[17], Ugur Ozcelik[12], Ana Reula[18-19], Jobst Roehmel[20], Christine van Gogh[21], Panayiotis Yiallouros[22-23], Soeren Marian Zappe[24], for the EPIC-PCD team*, Jean-François Papon[3,25]

Affiliations:

1. Institute of Social and Preventive Medicine, University of Bern, Switzerland.
2. Paediatric Respiratory Medicine, Children's University Hospital of Bern, University of Bern, Switzerland.
3. AP-HP, Hôpital Kremlin-Bicetre, Service d'ORL et de Chirurgie Cervico-Faciale, Le Kremlin-Bicêtre, France.
4. Faculté de Médecine, Université Paris-Saclay, Le Kremlin-Bicêtre, France.
5. Department of Otorhinolaryngology, Nicosia General Hospital, Nicosia, Cyprus.
6. Department of Otorhinolaryngology, and Primary Ciliary Dyskinesia Unit, La Fe University and Polytechnic Hospital, Valencia, Spain.
7. Medical School, Valencia University, Valencia, Spain.
8. AP-HP, Hôpital Henri Mondor et Centre Hospitalier Intercommunal de Créteil, Service d'Oto-Rhino-Laryngologie et de Chirurgie Cervico-Faciale, Créteil, France.
9. Department of Paediatrics, University Hospital, Leuven, Belgium.
10. Primary Ciliary Dyskinesia Centre, Southampton Children's Hospital, Southampton NHS Foundation Trust, Southampton, UK.
11. Faculté de Médecine, Université Paris-Est Créteil, Institut National de la Santé et de la Recherche Médicale, Centre National de la Recherche Scientifique, Hôpital Henri Mondor, Créteil, France.
12. Department of Pediatric Pulmonology, Hacettepe University, School of Medicine, Ankara, Turkey.
13. Department of Pediatric Pulmonology, Marmara University, School of Medicine, Istanbul, Turkey.
14. Department of pediatric pulmonology, Emma Children's Hospital, Amsterdam UMC, Vrije Universiteit, Amsterdam, The Netherlands.
15. Primary Ciliary Dyskinesia Centre, NIHR Respiratory Biomedical Research Centre, University of Southampton, Southampton, UK.

For peer review only

16. Department of Otorhinolaryngology, Head and Neck Surgery, University Hospital of Bern, University of Bern, Switzerland.
17. Department of Respiratory Diseases, University Hospital, Leuven, Belgium.
18. Biomedical Sciences Department, CEU-Cardenal Herrera University, Castellón, Spain.
19. Molecular, Cellular and Genomic Biomedicine Group, IIS La Fe, Valencia, Spain.
20. Department of Pediatric Pulmonology, Immunology and Critical Care Medicine, Charité-Universitätsmedizin, Berlin, Germany.
21. Department of Otolaryngology/ Head and Neck Surgery, Amsterdam University Medical Center, Amsterdam, The Netherlands.
22. Medical School, University of Cyprus, Nicosia, Cyprus.
23. Pediatric Pulmonology Unit, Hospital 'Archbishop Makarios III', Nicosia, Cyprus.
24. Department of Otorhinolaryngology, Head and Neck Surgery, Charité-Universitätsmedizin, Berlin, Germany.
25. Faculté de Médecine, Université Paris-Saclay, Le Kremlin-Bicêtre, France.

Correspondence: Myrofora Goutaki, Institute of Social and Preventive Medicine, University of Bern, Mittelstrasse 43, 3012 Bern, Switzerland. myrofora.goutaki@ispm.unibe.ch ; Tel.: +41 31 631 5973

***For the EPIC-PCD team** (listed in alphabetical order):
Dilber Ademhan (Hacettepe University, Turkey), Mihaela Alexandru (AP-HP, France), Andreas Anagiotos (Nicosia General Hospital, Cyprus), Miguel Armengot (La Fe University and Polytechnic Hospital, Spain), Emilie Bequignon (AP-HP, France), Irma Bon (Vrije Universiteit, The Netherlands), Mieke Boon (University Hospital Leuven, Belgium), Marina Bullo (University of Bern, Switzerland), Andrea Burgess (University of Southampton, UK), Carmen Casaulta (University of Bern, Switzerland), Marco Caversaccio (University of Bern, Switzerland), Nathalie Caversaccio (University of Bern, Switzerland), Andre Coste (AP-HP, France), Bruno Crestani (RESPIRARE, France), Sandra Diepenhorst (Vrije Universiteit, The Netherlands), Nagehan Emiralioglu (Hacettepe University, Turkey), Ela Erdem (Marmara University, Turkey), Pinar Ergenekon (Marmara University, Turkey), Nathalie Feyaerts (University Hospital Leuven, Belgium), Gabriel Georgiou (Nicosia General Hospital, Cyprus), Amy Glen (University of Southampton, UK), Christine van Gogh (Vrije Universiteit, The Netherlands), Yasemin Gokdemir (Marmara University, Turkey), Myrofora Goutaki (University of Bern, Switzerland), Onder Gunaydın (Hacettepe University, Turkey), Eric G. Haarman (Vrije Universiteit, The Netherlands), Amanda Harris (University of Southampton, UK), Sara-Lynn Hool (University of Bern, Switzerland), Isabelle Honoré (RESPIRARE, France), Hasnaa Ismail-Koch (University of Southampton, UK), Bulent Karadag (Marmara University, Turkey), Elisabeth Kieninger (University of Bern, Switzerland), Sookyung Kim (AP-HP, France), Panayiotis Kouis (University of Cyprus, Cyprus), Bernard Maitre (RESPIRARE, France), David Montani (RESPIRARE, France), Yin Ting Lam (University of Bern, Switzerland), Philipp Latzin (University of Bern, Switzerland), Marie Legendre (RESPIRARE, France), Natalie Lorent (University Hospital Leuven, Belgium), Jane S. Lucas (University of Southampton, UK), Alison McEvoy (University of Southampton, UK), Rana Mitri-Frangieh (RESPIRARE, France), Loretta Müller (University of Bern, Switzerland), Noelia Muñoz (La Fe University and Polytechnic Hospital, Spain), Ugur Ozcelik (Hacettepe University, Turkey), Beste Ozsezen (Hacettepe University, Turkey), Samantha Packham (University of Southampton, UK), Jean-François Papon (AP-HP, France), Ana Reula (La Fe University, Spain), Jobst Roehmel (Charité-

For peer review only - http://bmjopen.bmj.com/site/about/guidelines.xhtml

Universitätsmedizin Berlin, Germany), Simone Tanner (Vrije Universiteit, The Netherlands), Guillaume Thouvenin (RESPIRARE, France), Woolf T. Walker (University of Southampton, UK), Hannah Wilkins (University of Southampton, UK), Panayiotis Yiallouros (University of Cyprus, Cyprus), Soeren Marian Zappe (Charité-Universitätsmedizin Berlin, Germany).

For peer review only

For peer review only - http://bmjopen.bmj.com/site/about/guidelines.xhtml

Abstract

Introduction: Primary Ciliary Dyskinesia (PCD) is a rare, genetic, multiorgan disease with an estimated prevalence of 1 in 10,000. It affects mainly the upper and lower airways due to impaired mucociliary clearance. Almost all patients have sinonasal or otologic (ear-nose-throat, ENT) problems although the ENT clinical phenotype may present great variability. Despite that, data on PCD ENT manifestations are scarce and based on small single-centre studies. To date we know little about the spectrum and severity of PCD ENT disease, its association with lung disease, its course over life, and its determinants of prognosis.

This study protocol describes the aims and methods of the first prospective, observational, multinational cohort study focusing on ENT disease in PCD patients.

Methods and Analysis: The ENT Prospective International Cohort of PCD patients (EPIC-PCD) is a prospective standardised observational clinical cohort set up as a multinational multi-centre study, embedded into routine patient care. It aims to longitudinally characterise ENT disease in PCD patients and its association with lung disease, and to identify determinants of its prognosis. Patients of all ages, diagnosed with PCD who undergo an ENT clinical assessment at least once a year at one of the participating centres will be invited to participate. Collected data include diagnostic test results, results of ENT examinations, lung function measurements, information on management of ENT disease and patient-reported data on clinical symptoms and health-related quality of life (QoL). Data are collected using the standardised PCD-specific FOLLOW-PCD form and the validated QoL-PCD questionnaire.

Ethics and dissemination: The study has been reviewed and approved by the Human Research Ethics Committees at all participating centres, based on local legislation. The results of the study will be published in scientific journals, presented at scientific conferences and disseminated to participants and national patient organizations.

For peer review only - http://bmjopen.bmj.com/site/about/guidelines.xhtml

The study is registered at ClinicalTrials.gov (NCT04611516).

Keywords: Primary Ciliary Dyskinesia, Orphan disease, Epidemiology, Cohort study, Upper airways, rhinology, otology

For peer review only

For peer review only - http://bmjopen.bmj.com/site/about/guidelines.xhtml

Strengths and limitations of this study

- The EPIC-PCD is the first prospective international multicentre clinical study focusing on upper airways disease in patients with PCD and is embedded in routine patient care, allowing recruitment of large numbers of patients, which is crucial in rare diseases research.
- The study combines objective assessments of the upper airways and patient reported information on symptoms and health-related quality of life, all collected with disease-specific tools, in a standardised way.
- All participating centres are reference centres for PCD and have already established collaboration through previous and ongoing research initiatives.
- Although the study includes both paediatric and adult patients with PCD, all participants are followed up at established PCD clinical centres, therefore patients with atypical symptoms who remain undiagnosed or live in areas where diagnosis and care of PCD is not well established will not be included.
- The EPIC-PCD has the potential to become a valuable research resource and to help understanding better the disease in order to improve diagnosis, clinical care and quality of life of patients with PCD.

For peer review only - http://bmjopen.bmj.com/site/about/guidelines.xhtml

Introduction

Primary ciliary dyskinesia (PCD) is a rare genetic, multiorgan disorder affecting approximately 1:10,000 individuals.[1 2] Mutations in disease causing-genes result in defects in ciliary structure or function, leading to a wide range of symptoms from different organ systems.[3-5] The clinical phenotype of the disease is heterogeneous, however reduced airway mucociliary clearance predisposes almost all affected individuals to recurrent upper and lower respiratory infections.[6 7]

Persistent symptoms from the upper airways and ears (ear nose throat: ENT) are a common characteristic of most PCD patients. Ear symptoms commonly present in children with PCD as severe and bilateral otitis media with effusion (OME).[8-11] OME usually resolves spontaneously in otherwise healthy children, but in PCD it seems to persist longer and might need specific management.[8 12-15] In some cases, it may lead to hearing impairment and cause delays in speech development.[16 17] While otologic involvement seems to improve with age in PCD patients, sinonasal disease appears to persist and may worsen in adulthood.[18] First manifestations appear sometimes soon after birth and are usually nonspecific in the form of rhinitis and episodic facial pain due to recurrent infections leading gradually to chronic rhinosinusitis (CRS).[19-23] In adults, a recent study identified sinonasal disease as a primary cause of decreased quality of life in PCD patients.[18] Hypoplasia and agenesis of paranasal sinuses have also been described, while nasal polyps are more common in PCD patients compared to the general population, although this complication has not been well characterised. [18 24 25]

Despite being so frequent and impairing patients' physical health and quality of life (QoL), data on ENT disease in PCD are still scarce. Several large national and multinational research collaborations have made important advances in PCD research in the recent years, though most clinical studies focus on improving diagnosis and characterising lung disease.[26-29] Only few retrospective and single-centre studies have described ENT manifestations is some detail. Most studies focused on children and little is known about age-related changes,[8 13] while studies including standardised sinonasal and otologic evaluations were rare. We lack knowledge about the spectrum and severity of ENT disease

For peer review only - http://bmjopen.bmj.com/site/about/guidelines.xhtml

in patients with PCD, its association with lung disease, its course over life, and determinants of prognosis.

This study protocol describes the aims and methods of the first prospective, observational, multinational cohort study focusing on ENT disease in PCD patients.

Study objectives

This study aims to characterise ENT disease in PCD patients and its association with lung disease, and to identify determinants of its course and prognosis. Specifically, we aim to:

a) Assess the prevalence and severity of sinonasal and otologic symptoms and the frequency and range of signs and physiological findings assessed during standardised ENT physical examination, and describe possible differences by age;

b) Study the association of sinonasal and otologic disease with lung disease in PCD patients;

c) Identify determinants of the course and prognosis of sinonasal and otologic disease in PCD patients.

Methods and analysis

Study design

The ENT Prospective International Cohort of PCD patients (EPIC-PCD) is a prospective observational clinical cohort set up as a multinational multi-centre study. It is embedded into routine patient care of participating reference centres for PCD and patients continue to be managed according to local procedures and protocols. The study is hosted at the Institute of Social and Preventive Medicine (ISPM) (EPIC-PCD data centre) at the University of Bern, Switzerland, and is managed in collaboration with participating centres (EPIC-PCD clinics) (ClinicalTrials.gov Identifier: NCT04611516).

Patient data are collected prospectively. Baseline information is collected at the enrolment of the patient in the study and follow-up data during clinical follow-up visits at the participating centres. The study has no foreseen endpoint. Patient follow-up is planned for a minimum period of 4 years, however it will be extended if collaborators agree.

For peer review only - http://bmjopen.bmj.com/site/about/guidelines.xhtml

Study population

Inclusion criteria and participating centres

Patients of all ages, diagnosed with PCD, according to the European Respiratory Society (ERS) guidelines, and followed-up at one of the participating centres are invited to participate to the EPIC-PCD.[26] Patients are excluded if they do not undergo an ENT clinical assessment, at least once a year during the study period.

Patient recruitment has started at the following centres (EPIC-PCD clinics; Figure 1):

- University Hospital Bicêtre Paris-Sud, France;
- Centre Hospitalier Intercommunal Creteil and University Hospital Henri Mondor, France;
- Hospital Universitario La Fe in Valencia, Spain;
- University Hospital of Southampton, United Kingdom;
- University Hospital of Leuven, Belgium;
- VU University medical center (VUmc) in Amsterdam, The Netherlands;
- University of Cyprus;
- Hacettepe University in Ankara, Turkey;
- Marmara University in Istanbul, Turkey;
- University Children´s Hospital Charité-Universitätsmedizin in Berlin, Germany, and
- University Children's hospital in Bern Switzerland.

After the establishment of the study in these initial EPIC-PCD clinics, we will invite more PCD centres to join the study and begin patient recruitment.

Study procedures

Patient identification and recruitment

Eligible patients are identified by the local clinical team at the EPIC-PCD clinics and are invited to participate in the study, receiving all study information. Patients are recruited during a clinical follow-

For peer review only - http://bmjopen.bmj.com/site/about/guidelines.xhtml

up visit; there might be small variations in recruitment and data collection between participating centres to allow the study to be embedded in the regular clinical routine.

Clinical assessments

Eligible patients are followed regularly at the EPIC-PCD clinics, at 3 to 6-month intervals, as planned for clinical reasons, regardless of their participation in the study. Each patient undergoes a detailed sinonasal and otologic examination by ENT specialists, at minimum once a year, during a scheduled follow-up visit. Additional ENT examinations are performed upon clinical indication in-between follow-up visits. Patients will not be subjected to additional invasive measurements solely for the purposes of the study; examinations and tests are only done if clinically indicated or as part of the local follow-up protocol. All examinations and tests are performed following the local Standard Operating Procedures (SOPs) of the clinic. The extended study team discusses differences and ensures that collected data are comparable.

Data collection instruments

For the collection of patient data, we use standardised PCD-specific instruments, specifically FOLLOW-PCD and QoL-PCD.

FOLLOW-PCD is a disease-specific form for standardised prospective data collection during routine clinical follow-up of PCD patients.[30] It was developed by a multidisciplinary, international working group of the BEAT-PCD COST Action network (Better Experimental Approaches to Treat PCD).[27 31] FOLLOW-PCD has a modular structure, which permits different members of the clinical team (pulmonologists, ENT physicians, diagnostic experts, physiotherapists, lung function technicians etc.) to complete different modules. FOLLOW-PCD consists of seven modules that enable an extensive evaluation at baseline and annual reviews, when all modules are completed, and a shorter evaluation at routine (e.g. 3-monthly) follow-up, where only a few modules are used. For the EPIC-PCD study, we use specific modules to collect clinical data relevant to the study, particularly demographic and

For peer review only - http://bmjopen.bmj.com/site/about/guidelines.xhtml

diagnostic information, results of the physical ENT examination, results of clinical tests and data on hospitalizations and treatments related to the upper airways.

Some modules of FOLLOW-PCD have been developed into patient questionnaires (FOLLOW-PCD questionnaire) that can be completed by the patients during each clinical follow-up visit. There are three age-specific versions of the questionnaires: one for adult patients, one for adolescents 14-18 years old and one for the parents or caretakers of patients aged less than 14 years. The FOLLOW-PCD questionnaire has been originally developed in English, German and Greek and is translated in additional languages using a standardised procedure.

In addition to the FOLLOW-PCD questionnaire, we will ask the patients to complete once a year the **QoL-PCD**, a disease-specific, health-related quality of life instrument for PCD, validated and translated in several languages. [32-35] There are four different versions available for a) children (6-12 years), b) adolescents (13-17 years), c) adults (≥18 years), and d) a parent-proxy measure for children ≤12 years. The different versions of the questionnaire are divided into 7-10 hypothesised scales generated by multitrait analysis. All versions include two scales about upper respiratory symptoms, and ears and hearing symptoms, which are particularly relevant to this project. The QoL-PCD has demonstrated good internal consistency and test-retest reliability, and is a valuable tool for observational and interventional studies in PCD.

The FOLLOW-PCD questionnaire, does not address QoL, therefore the two questionnaires provide important complimentary information. Both questionnaires have been translated inthe languages of the participating EPIC-PCD clinics.

Lastly, some centres include as part of their regular patient follow-up collection of information on sinusitis-related QoL using the Sino-nasal Outcome Test-22 (SNOT-22) questionnaire.[36]

What information is collected

Figure 2 provides a brief overview of the information collected from participating patients at the baseline and follow-up visits, including the minimum required data, which are assessed in all participating centres and additional data which will be collected if available at the specific centre and

For peer review only - http://bmjopen.bmj.com/site/about/guidelines.xhtml

patient visit. Small exemptions are allowed when part of the minimum required data have not been collected because an examination was not performed due to e.g. the patient's age, as long as each patient undergoes a specialised ENT examination at least once a year.

ENT examinations

The ENT examinations performed in the participating centres in addition to physical examination include nasal endoscopy (either rigid or flexible), nasal function tests (e.g. patency, olfaction) tests, tympanometry, audiometry, and auditory brainstem evoked response. Nasal endoscopy, tympanometry and audiometry are performed regularly at all centres during follow-up; the remaining tests are performed only in selected, specialised centres either at initial diagnosis or during follow-up. When clinically indicated, patients will undergo computed tomography of the sinuses or the ears. When available, we will collect data of previous imaging examinations of the sinuses or the ears from clinical records.

Other data from clinical records

At recruitment, baseline medical information and results of diagnostic tests will be extracted from clinical records and recorded using FOLLOW-PCD. In addition to the medical data from the upper airways, we collect data from lung function measurements including spirometry and when available multiple breath-washout measurements and lung imaging. This information will allow us to assess associations between upper and lower respiratory disease in PCD.

Upon indication, nasopharyngeal or nasal swab samples, and when possible middle meatal samples, and sputum or cough samples for microbiological cultures and results will be collected. In case of otorrhea, ear drainage samples will also be taken. We also collect information related to hospitalisations and surgical procedures related to upper airways disease and data on upper airway management.

For peer review only

Information from questionnaires

Participating patients are asked to complete the FOLLOW-PCD and QoL-PCD questionnaires described earlier. The FOLLOW-PCD questionnaire includes sections on upper and lower respiratory symptoms, health-related behaviours (e.g. exercise, smoking) and environmental factors. The QoL-PCD questionnaire assesses how the disease affects the quality of life of PCD patients.

Study database

All collected data are entered into a secure web-based database using the database program Research Electronic Data Capture (REDCap) hosted by the Clinical Trials Unit (CTU) Bern.[37] REDCap is widely used in academic research and allows data entry and extraction in various formats. Contributing centres are responsible for the pseudonymisation of the data contributed to the study. Only limited research staff from the EPIC-PCD data team has access to the whole dataset; local investigators at the EPIC-PCD clinics have access only to data from their clinic (text box 1). Data generation, transmission, storage and analysis of health-related personal data within the study follow the Swiss legal requirements for data protection. All study team members are trained on data protection and dealing with particularly sensitive data.

Study power and sample size

We expect to recruit at least 450-550 patients in EPIC-PCD. This number is based on the study feasibility and the number of patients followed-up in the EPIC-PCD clinics and is considered large enough for a prospective study in such a rare disease and much larger than any other available PCD dataset on ENT disease. After the establishment of the cohort, it is now possible for more centres to join and contribute patients, resulting in even higher numbers of patients. The planned analyses are up to a point exploratory and do not follow specific hypotheses, although we have few assumptions based on the limited literature. This does not allow for a precise power calculation; however we will ensure that each performed analysis has sufficient power to draw meaningful conclusions.

For peer review only - http://bmjopen.bmj.com/site/about/guidelines.xhtml

Text Box 1: Contributing to and accessing data in the ENT Prospective International Cohort of PCD patients (EPIC-PCD)

How to contribute data

- Centres that wish to participate in the project and contribute data can contact the EPIC-PCD data team. As soon as ethical approval is obtained and centre representatives have signed a data delivery agreement, they will receive a password to access the online software REDCap and they will be able to enter their data directly prospectively. We offer information and technical support wherever needed.

How to access data

- Participating centres keep constant access to their datasets and can export them directly in various formats for local analyses. Participating centres may also extract their datasets and contribute relevant data to other multinational projects or to the international PCD registry.
- The use of EPIC-PCD data from several centres for studies and publications is regulated by the EPIC-PCD committee, which consists of members of the EPIC-PCD data centre and representatives of the EPIC-PCD clinics. A series of publications are originally planned to be led by the EPIC-PCD data team, as part of the research grant funding the set-up and management of the study.
- For additional publications, collaborators should submit a concept sheet describing the planned analysis and publication to the committee, which will approve or oppose the planned publication and use of data. If the planned analysis is approved, a publication agreement will be signed and the EPIC-PCD data centre will prepare a partial dataset for the proposed analysis and will work closely with the lead researchers of each planned study for methodological input and support.
- For further details, contact: myrofora.goutaki@ispm.unibe.ch

REDCap: Research Electronic Data Capture.

For peer review only - http://bmjopen.bmj.com/site/about/guidelines.xhtml

Planned analysis and outcomes of interest

First, we will describe prevalence and severity of ENT problems, stratified by age, age at diagnosis, sex, country, ciliary ultrastructural defect or genetic mutation. Outcomes of interest will be sinonasal and otologic symptoms and signs (e.g. rhinitis, loss of smell, sinusitis, otitis, impaired hearing) and the results of ENT examinations (mainly of nasal endoscopy, tympanometry, audiometry and when available imaging). We will estimate prevalence of binary outcomes and mean values of continuous outcomes by using constant-only models. We will use logistic regression models for binary outcomes, and linear models for continuous outcomes. Associations with determinants will be assessed in multivariable mixed models.

As a second step, we will assess the association of baseline ENT problems with lung disease progression expressed in terms of lung function tests, sputum cultures and, if available, lung imaging results. For this analysis, lung function will be the main outcome, measured primarily with spirometry and plethysmography measuring forced expiratory vital capacity (FVC) and forced expiratory volume in 1 second (FEV1). Data will be converted into z-scores adjusted for sex, height, age, and ethnicity, using the Global Lung Initiative (GLI) reference values. In some centres, PCD patients also perform multiple breath washout measurements at follow-up, which have been reported to be more sensitive in detecting early lung disease. In those patients, lung clearance index (LCI) will be considered as a secondary outcome, if patients´ numbers allow it. We will use mixed models, including the baseline ENT disease characteristics with an interaction of each with age as fixed effects and the patients and the centres as random effects.

Finally, we will analyse follow-up data on ENT symptoms and examinations to describe the short-term course of sinonasal and otologic disease during a 2-year follow-up, and identify determinants of disease prognosis. Here, outcomes of interest will be the severity of CRS (based on a symptoms score), loss of smell, bacterial colonization of the upper respiratory tract, need of surgical management of the sinuses, recurrent otitis media, otitis media with effusion, hearing impairment, and need of surgical management of the ears. We will first describe how the outcomes change over

For peer review only - http://bmjopen.bmj.com/site/about/guidelines.xhtml

the follow-up time in binomial or multinomial (for outcomes with more than three categories such as hearing impairment) mixed models, using separate models for each outcome. Then, we will study determinants of disease prognosis categorising patients in four groups for each outcome based on the evaluation at baseline and at 2-year follow-up: a) patients who never reported the outcome, b) patients negative for the outcome at baseline but positive at follow-up, c) patients positive at baseline but negative at follow-up and d) patients positive at both time points. We will use mixed-effects multinomial regression models, including all possible determinants as fixed effects. We will select possible determinants from characteristics available at baseline including symptoms and measurements; age at diagnosis, ultrastructural defects, and genetic mutations; and general patient characteristics such as age and sex. We will make our determinants selection based on directed acyclic graphs, considering existing literature. Data will be collected prospectively and in a standardised way resulting in small data heterogeneity between participating centres, however we will still need to account for possible differences between countries in all analyses. Thus, we will analyse all outcomes of interest using random-effects models that account for clustering of patients within participating centres.

For all analyses, results will be interpreted with caution considering the clinical importance and not just mere statistically significant findings. If needed, we will correct for multiple testing. All missing values will be described in detail, however we expect this will not be an issue for the important variables as the data is collected prospectively. In case we have significant amount of missing data, we will consider subgroup analyses or multiple imputation of missing data to deal with this issue, depending on what is the most appropriate approach for the specific question and analysis.

Ethics and dissemination

Ethical approval to share pseudo-anonymised patient data was obtained locally at each participating centre. Written informed consent will be obtained in accordance with the national/local data protection laws, from patients aged 14 years or more (might differ per country based on local laws)

For peer review only - http://bmjopen.bmj.com/site/about/guidelines.xhtml

or from the patient's parents/caregivers for younger patients. Patients can withdraw their consent and their data from the study at any time by contacting the clinical team at each participating centre.

The results of the study will be published in scientific journals and presented at scientific conferences to raise awareness about upper respiratory disease in PCD patients.

The principal investigator of the study affirms and upholds the principle of the participants' right to dignity, privacy and health and that the project team shall comply with applicable privacy laws. Especially, anonymity of the participants shall be guaranteed when presenting the data at scientific meetings or publishing them in scientific journals. Individual participant medical information obtained during this research project is considered confidential and will not be disclosed to third parties. Data will not be deposited at an open access repository. Because of the rarity of the disease although data will be pseudonymised, it may include still sensitive information, therefore participants will not be asked to give consent to have their data deposited publicly.

Patient and public involvement

Several patient support organizations were consulted and support the study. Additionally, the study team works closely with a team of PCD patients who consult the BEAT-PCD clinical research collaboration (CRC) network to ensure that all studies support their interests[38]. Summaries of results in lay language will be disseminated to participants and national patient organizations by the EPIC-PCD data centre and the EPIC-PCD clinics and to the relevant stakeholders and the public through the BEAT-PCD CRC website and social media.

Conclusion

The EPIC-PCD is the first multicentre prospective cohort study aiming to characterise phenotypically the ENT disease in PCD and its associations with the lower airways. It recruits patients from all age groups from 11 centres from nine countries and more centres could join in the future. The main strength of the study is the large number of patients planned to be recruited and the inclusion of prospective standardised data from ENT clinical examinations and tests. Another strength is the

For peer review only - http://bmjopen.bmj.com/site/about/guidelines.xhtml

For peer review only

collection of detailed standardised patient reported data on clinical symptoms, lifestyle and quality of life. The cohort is nested in clinical care allowing recruiting of large numbers of patients and using data routinely collected in clinics. All participating centres are reference centres for PCD, with already established collaboration through previous and ongoing PCD research initiatives, following evidence-based guidelines for the diagnosis of PCD[26].

The need for research projects focusing on ENT aspects has been repeatedly noted during past PCD research initiatives. The EPIC-PCD is set up and can be further developed in the framework of the BEAT-PCD CRC supported by the European Respiratory Society[38]. The BEAT-PCD CRC is a large multidisciplinary network, which includes more than 500 researchers and clinicians from more than 30 countries. The EPIC-PCD is part of the BEAT-PCD research portfolio, which ensures widespread dissemination of the study results to researchers, clinicians, patients and all relevant stakeholders. The EPIC-PCD has the potential to become a valuable research resource and to help understanding better the disease in order to improve diagnosis, genetic counselling, clinical care and quality of life of patients with PCD.

For peer review only

For peer review only - http://bmjopen.bmj.com/site/about/guidelines.xhtml

Authors' contributions: MG designed the study. MG, YTL and JFP drafted the protocol. All authors critically revised and approved the manuscript.

Funding statement: This work was supported by the Swiss National Science Foundation grant number (SNFPZ00P3_185923). Future grant applications by all partners to different funding bodies will provide continuous support. Authors participate in the BEAT-PCD clinical research collaboration, supported by the European Respiratory Society and most participating centres are part of the ERN-LUNG (European Reference Network) PCD-Core.

Competing interests statement: The authors have no conflict of interest related to this study. Dr. Latzin reports personal fees from Gilead, Novartis, OM pharma, Polyphor, Roche, Santhera, Schwabe, Vertex, Vifor, Zambon and grants from Vertex, all outside the submitted work.

Acknowledgements: We thank all the patients with PCD in the cohort and their families, and the PCD support organisations (especially: PCD Family Support Group UK; Association ADCP France; Kartagener Syndrom und Primäre Ciliäre Dyskinesie e. V. Deutschland/ Deutschschweiz) for their close collaboration. We also thank all the researchers in the participating centres who are involved in recruitment, data collection and data entry, and work closely with us through the whole process of participating to the EPIC-PCD.

For peer review only

References

1. Kuehni CE, Frischer T, Strippoli MP, et al. Factors influencing age at diagnosis of primary ciliary dyskinesia in European children. *The European respiratory journal* 2010;36(6):1248-58. doi: 10.1183/09031936.00001010 [published Online First: 2010/06/10]
2. Lucas JS, Walker WT, Kuehni CE, et al. Primary Ciliary Dyskinesia. *In: Courdier J-F, editor Orphan Lung diseases European Respiratory Monograph* 2011:201-17.
3. Kennedy MP, Omran H, Leigh MW, et al. Congenital heart disease and other heterotaxic defects in a large cohort of patients with primary ciliary dyskinesia. *Circulation* 2007;115(22):2814-21. doi: 10.1161/CIRCULATIONAHA.106.649038 [published Online First: 2007/05/23]
4. Shapiro AJ, Davis SD, Ferkol T, et al. Laterality defects other than situs inversus totalis in primary ciliary dyskinesia: insights into situs ambiguus and heterotaxy. *Chest* 2014;146(5):1176-86. doi: 10.1378/chest.13-1704 [published Online First: 2014/03/01]
5. Vanaken GJ, Bassinet L, Boon M, et al. Infertility in an adult cohort with primary ciliary dyskinesia: phenotype-gene association. *The European respiratory journal* 2017;50(5) doi: 10.1183/13993003.00314-2017 [published Online First: 2017/11/11]
6. Goutaki M, Meier AB, Halbeisen FS, et al. Clinical manifestations in primary ciliary dyskinesia: systematic review and meta-analysis. *The European respiratory journal* 2016;48(4):1081-95. doi: 10.1183/13993003.00736-2016 [published Online First: 2016/08/06]
7. Stannard W, O'Callaghan C. Ciliary function and the role of cilia in clearance. *Journal of aerosol medicine : the official journal of the International Society for Aerosols in Medicine* 2006;19(1):110-5. doi: 10.1089/jam.2006.19.110 [published Online First: 2006/03/23]
8. Pruliere-Escabasse V, Coste A, Chauvin P, et al. Otologic features in children with primary ciliary dyskinesia. *Archives of otolaryngology--head & neck surgery* 2010;136(11):1121-6. doi: 10.1001/archoto.2010.183 [published Online First: 2010/11/17]
9. Sommer JU, Schafer K, Omran H, et al. ENT manifestations in patients with primary ciliary dyskinesia: prevalence and significance of otorhinolaryngologic co-morbidities. *European archives of oto-rhino-laryngology : official journal of the European Federation of Oto-Rhino-Laryngological Societies (EUFOS) : affiliated with the German Society for Oto-Rhino-Laryngology - Head and Neck Surgery* 2011;268(3):383-8. doi: 10.1007/s00405-010-1341-9 [published Online First: 2010/07/24]
10. el-Sayed Y, al-Sarhani A, al-Essa AR. Otological manifestations of primary ciliary dyskinesia. *Clinical otolaryngology and allied sciences* 1997;22(3):266-70. [published Online First: 1997/06/01]
11. Takeuchi K, Kitano M, Sakaida H, et al. Analysis of Otologic Features of Patients With Primary Ciliary Dyskinesia. *Otology & neurotology : official publication of the American Otological Society, American Neurotology Society [and] European Academy of Otology and Neurotology* 2017;38(10):e451-e56. doi: 10.1097/MAO.0000000000001599 [published Online First: 2017/11/15]
12. Berman S. Otitis media in children. *The New England journal of medicine* 1995;332(23):1560-5. doi: 10.1056/NEJM199506083322307 [published Online First: 1995/06/08]
13. Majithia A, Fong J, Hariri M, et al. Hearing outcomes in children with primary ciliary dyskinesia—a longitudinal study. *International journal of pediatric otorhinolaryngology* 2005;69(8):1061-64.
14. Campbell RG, Birman CS, Morgan L. Management of otitis media with effusion in children with primary ciliary dyskinesia: a literature review. *International journal of pediatric otorhinolaryngology* 2009;73(12):1630-8. doi: 10.1016/j.ijporl.2009.08.024 [published Online First: 2009/10/03]
15. Majithia A, Fong J, Hariri M, et al. Hearing outcomes in children with primary ciliary dyskinesia--a longitudinal study. *International journal of pediatric otorhinolaryngology* 2005;69(8):1061-4. doi: 10.1016/j.ijporl.2005.02.013 [published Online First: 2005/07/12]

For peer review only - http://bmjopen.bmj.com/site/about/guidelines.xhtml

16. Kreicher KL, Schopper HK, Naik AN, et al. Hearing loss in children with primary ciliary dyskinesia. *International journal of pediatric otorhinolaryngology* 2018;104:161-65. doi: 10.1016/j.ijporl.2017.11.005 [published Online First: 2017/12/31]
17. Yoshinaga-Itano C, Sedey AL, Coulter DK, et al. Language of early- and later-identified children with hearing loss. *Pediatrics* 1998;102(5):1161-71. [published Online First: 1998/10/31]
18. Bequignon E, Dupuy L, Zerah-Lancner F, et al. Critical Evaluation of Sinonasal Disease in 64 Adults with Primary Ciliary Dyskinesia. *Journal of clinical medicine* 2019;8(5) doi: 10.3390/jcm8050619 [published Online First: 2019/05/10]
19. Hosie PH, Fitzgerald DA, Jaffe A, et al. Presentation of primary ciliary dyskinesia in children: 30 years' experience. *Journal of paediatrics and child health* 2015;51(7):722-6. doi: 10.1111/jpc.12791 [published Online First: 2014/12/17]
20. Morgan LC, Birman CS. The impact of Primary Ciliary Dyskinesia on the upper respiratory tract. *Paediatric respiratory reviews* 2016;18:33-8. doi: 10.1016/j.prrv.2015.09.006 [published Online First: 2016/02/24]
21. Noone PG, Leigh MW, Sannuti A, et al. Primary ciliary dyskinesia: diagnostic and phenotypic features. *Am J Respir Crit Care Med* 2004;169(4):459-67. doi: 10.1164/rccm.200303-365OC [published Online First: 2003/12/06]
22. Pifferi M, Bush A, Rizzo M, et al. Olfactory dysfunction is worse in primary ciliary dyskinesia compared with other causes of chronic sinusitis in children. *Thorax* 2018;73(10):980-82. doi: 10.1136/thoraxjnl-2017-210661 [published Online First: 2018/03/02]
23. Behan L, Dimitrov BD, Kuehni CE, et al. PICADAR: a diagnostic predictive tool for primary ciliary dyskinesia. *The European respiratory journal* 2016;47(4):1103-12. doi: 10.1183/13993003.01551-2015 [published Online First: 2016/02/27]
24. Campbell R. Managing upper respiratory tract complications of primary ciliary dyskinesia in children. *Current opinion in allergy and clinical immunology* 2012;12(1):32-8. doi: 10.1097/ACI.0b013e32834eccc6 [published Online First: 2011/12/14]
25. Pifferi M, Bush A, Caramella D, et al. Agenesis of paranasal sinuses and nasal nitric oxide in primary ciliary dyskinesia. *The European respiratory journal* 2011;37(3):566-71. doi: 10.1183/09031936.00068810 [published Online First: 2010/07/24]
26. Lucas JS, Barbato A, Collins SA, et al. European Respiratory Society guidelines for the diagnosis of primary ciliary dyskinesia. *The European respiratory journal* 2017;49(1) doi: 10.1183/13993003.01090-2016 [published Online First: 2016/11/12]
27. Rubbo B, Behan L, Dehlink E, et al. Proceedings of the COST action BM1407 inaugural conference BEAT-PCD: translational research in primary ciliary dyskinesia - bench, bedside, and population perspectives. *BMC proceedings* 2016;10(Suppl 9):66. doi: 10.1186/s12919-016-0067-0 [published Online First: 2017/08/17]
28. Goutaki M, Maurer E, Halbeisen FS, et al. The international primary ciliary dyskinesia cohort (iPCD Cohort): methods and first results. *The European respiratory journal* 2017;49(1) doi: 10.1183/13993003.01181-2016 [published Online First: 2017/01/06]
29. Halbeisen FS, Goutaki M, Spycher BD, et al. Lung function in patients with primary ciliary dyskinesia: an iPCD Cohort study. *The European respiratory journal* 2018;52(2) doi: 10.1183/13993003.01040-2018 [published Online First: 2018/07/28]
30. Goutaki M, Papon JF, Boon M, et al. Standardised clinical data from patients with primary ciliary dyskinesia (PCD): FOLLOW-PCD *ERJ Open Res Submitted* 2019
31. Gardner LE, Horton KL, Shoemark A, et al. Proceedings of the 4(th) BEAT-PCD Conference and 5(th) PCD Training School. *BMC proceedings* 2020;14(Suppl 8):7. doi: 10.1186/s12919-020-00191-3 [published Online First: 2020/06/25]
32. Dell SD, Leigh MW, Lucas JS, et al. Primary Ciliary Dyskinesia: First Health-related Quality-of-Life Measures for Pediatric Patients. *Annals of the American Thoracic Society* 2016;13(10):1726-35. doi: 10.1513/AnnalsATS.201603-198OC [published Online First: 2016/07/28]

For peer review only - http://bmjopen.bmj.com/site/about/guidelines.xhtml

33. Lucas JS, Behan L, Dunn Galvin A, et al. A quality-of-life measure for adults with primary ciliary dyskinesia: QOL-PCD. *The European respiratory journal* 2015;46(2):375-83. doi: 10.1183/09031936.00216214 [published Online First: 2015/05/16]
34. Behan L, Leigh MW, Dell SD, et al. Validation of a health-related quality of life instrument for primary ciliary dyskinesia (QOL-PCD). *Thorax* 2017;72(9):832-39. doi: 10.1136/thoraxjnl-2016-209356 [published Online First: 2017/03/02]
35. Behan L, Leigh MW, Dell SD, et al. Validation of paediatric health-related quality of life instruments for primary ciliary dyskinesia (QOL-PCD). *Pediatric Pulmonology (In Press)* 2019
36. Kennedy J, Hubbard M, Huyett P, et al. Sino-nasal outcome test (SNOT-22): a predictor of postsurgical improvement in patients with chronic sinusitis. *Ann Allergy Asthma Immunol* 2013;111(4):246-251.e2 doi: doi:10.1016/j.anai.2013.06.033
37. Harris PA, Taylor R, Thielke R, et al. Research electronic data capture (REDCap)--a metadata-driven methodology and workflow process for providing translational research informatics support. *Journal of biomedical informatics* 2009;42(2):377-81. doi: 10.1016/j.jbi.2008.08.010 [published Online First: 2008/10/22]
38. Goutaki M, Crowley S, Dehlink E, et al. The BEAT-PCD (Better Experimental Approaches to Treat Primary Ciliary Dyskinesia) Clinical Research Collaboration. *The European respiratory journal* 2021;57(2) doi: 10.1183/13993003.04601-2020 [published Online First: 2021/02/06]

Figure Legends

Figure 1: Centres currently participating to the EPIC-PCD cohort study.

The EPIC-PCD clinics are marked with a black dot and the EPIC-PCD data centre with a yellow dot.

Figure 2: Overview of the information collected during the study

For peer review only

For peer review only - http://bmjopen.bmj.com/site/about/guidelines.xhtml

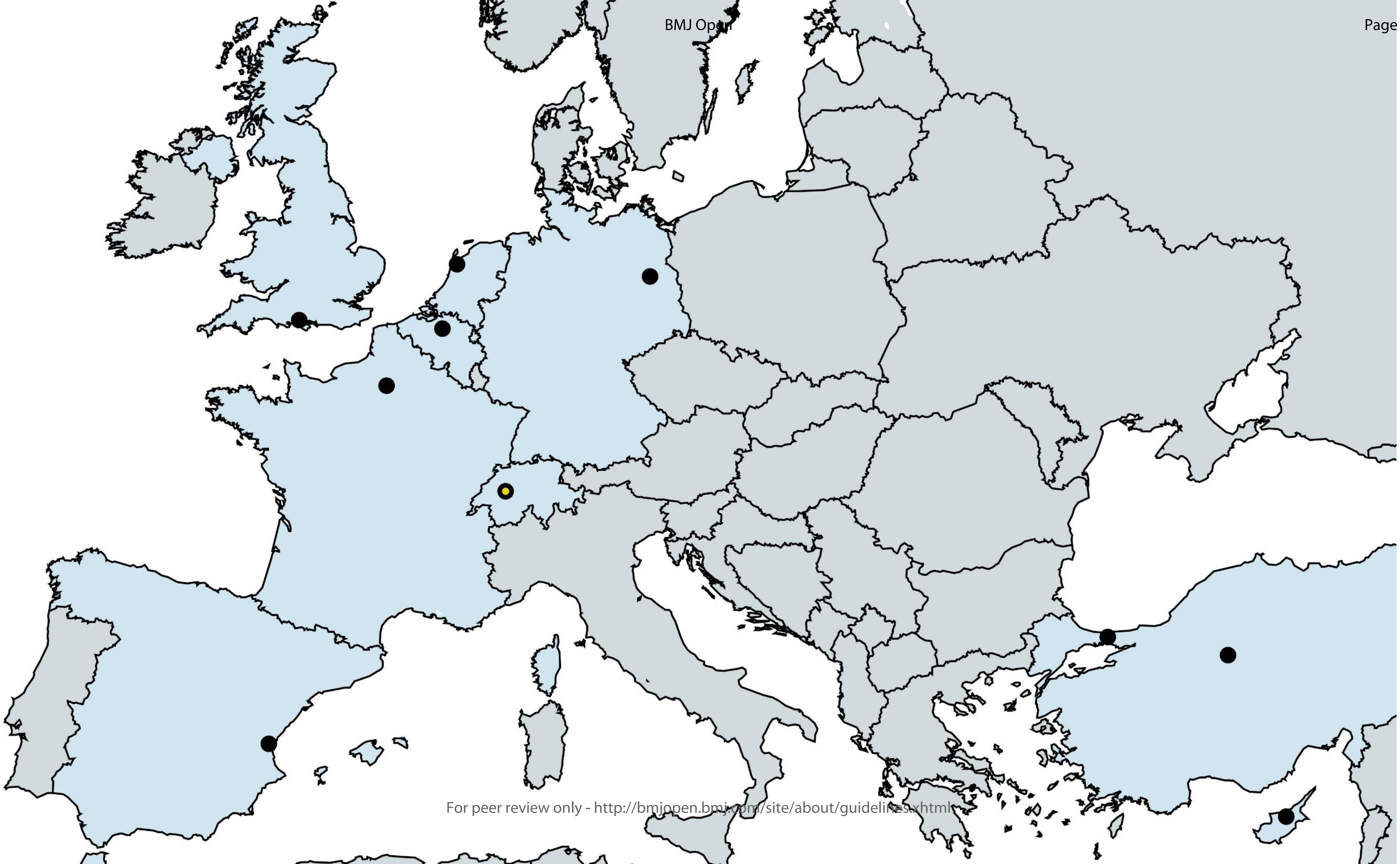

For peer review only - http://bmjopen.bmj.com/site/about/guidelines.xhtml

	BASELINE visit (patient recruitment)	FOLLOW-UP visits (at least once/year)
MINIMUM REQUIREMENTS	• FOLLOW-PCD patient questionnaire<br>• Standardised ENT examination including:<br>Audiometry<br>Tympanometry<br>Nasal endoscopy<br>• Spirometry<br>• Height/Weight<br>• Information on ENT management	• FOLLOW-PCD patient questionnaire<br>• Standardised ENT examination including:<br>Audiometry<br>Tympanometry<br>Nasal endoscopy<br>• Spirometry<br>• Height/Weight<br>• Information on ENT management
	• Diagnostic information<br>• Baseline medical history	
IF AVAILABLE	• QoL-PCD questionnaire/ SNOT-22<br>• Additional ENT examination results e.g.:<br>Nasal function tests/ Audiovestibular function tests/ Auditory brainstem evoked response/ Sinus, ear CT<br>• Plethysmography, Multiple breath washout<br>• Microbiology test results from upper and lower airways	• QoL-PCD questionnaire/ SNOT-22<br>• Additional ENT examination results e.g.:<br>Nasal function tests/ Audiovestibular function tests/ Auditory brainstem evoked response / Sinus, ear CT<br>• Plethysmography, Multiple breath washout<br>• Microbiology test results from upper and lower airways

BMJ Open

Study protocol: the Ear-Nose-Throat (ENT) Prospective International Cohort of patients with Primary Ciliary dyskinesia (EPIC-PCD)

Journal:	*BMJ Open*
Manuscript ID	bmjopen-2021-051433.R1
Article Type:	Protocol
Date Submitted by the Author:	14-Sep-2021
Complete List of Authors:	Goutaki, Myrofora ; University of Bern, Institute of Social and Preventive Medicine; University of Bern, Paediatric Respiratory Medicine, Children's University Hospital of Bern<br>Lam, Yin Ting; University of Bern, Institute of Social and Preventive Medicine<br>Alexandru, Mihaela; AP-HP, Hôpital Kremlin-Bicetre, Service d'ORL et de Chirurgie Cervico-Faciale; Université Paris-Saclay, Faculté de Médecine<br>Anagiotos, Andreas; Nicosia General Hospital, Department of Otorhinolaryngology<br>Armengot, Miguel; La Fe University and Polytechnic Hospital, Department of Otorhinolaryngology, and Primary Ciliary Dyskinesia Unit; University of Valencia, Medical School<br>Bequignon, Emilie; AP-HP, Hôpital Henri Mondor et Centre Hospitalier Intercommunal de Créteil, Service d'Oto-Rhino-Laryngologie et de Chirurgie Cervico-Faciale<br>Boon, Mieke; KU Leuven University Hospitals Leuven, Department of Paediatrics<br>Burgess, Andrea; Southampton NHS Foundation Trust, Primary Ciliary Dyskinesia Centre, Southampton Children's Hospital<br>Coste, Andre; AP-HP, Hôpital Henri Mondor et Centre Hospitalier Intercommunal de Créteil, Service d'Oto-Rhino-Laryngologie et de Chirurgie Cervico-Faciale; Université Paris-Est Créteil Val de Marne Faculté de médecine, Institut National de la Santé et de la Recherche Médicale, Centre National de la Recherche Scientifique, Hôpital Henri Mondor<br>Emiralioglu, Nagehan; Hacettepe University Faculty of Medicine, Department of Pediatric Pulmonology<br>Erdem, Ela; Marmara University School of Medicine, Department of Pediatric Pulmonology<br>Haarman, Eric G.; Vrije Universiteit Amsterdam, Department of pediatric pulmonology, Emma Children's Hospital, Amsterdam UMC<br>Harris, Amanda; University of Southampton, Primary Ciliary Dyskinesia Centre, NIHR Respiratory Biomedical Research Centre<br>Hool, Sara-Lynn; University of Bern, Department of Otorhinolaryngology, Head and Neck Surgery, University Hospital of Bern<br>Karadag, Bulent; Marmara University School of Medicine, Department of Pediatric Pulmonology<br>Kim, Sookyung; AP-HP, Hôpital Kremlin-Bicetre, Service d'ORL et de Chirurgie Cervico-Faciale

	Latzin, Philipp; University of Bern, Paediatric Respiratory Medicine, Children’s University Hospital of Bern<br>Lorent, Natalie; KU Leuven University Hospitals Leuven, Department of Respiratory Diseases<br>Ozcelik, Ugur; Hacettepe University Faculty of Medicine, Department of Pediatric Pulmonology<br>Reula, Ana; CEU Cardenal Herrera University, Biomedical Sciences Department; IIS La Fe, Molecular, Cellular and Genomic Biomedicine Group<br>Roehmel, Jobst; Charité Universitätsmedizin Berlin, Department of Pediatric Pulmonology, Immunology and Critical Care Medicine<br>van Gogh, Christine; Amsterdam University Medical Centres, Department of Otolaryngology/ Head and Neck Surgery<br>Yiallouros, Panayiotis; University of Cyprus, Medical School; Pediatric Pulmonology Unit, Hospital ‘Archbishop Makarios III’<br>Zappe, Soeren; Charité Universitätsmedizin Berlin, Department of Otorhinolaryngology, Head and Neck Surgery<br>PAPON, Jean; AP-HP, Hôpital Kremlin-Bicetre, Service d’ORL et de Chirurgie Cervico-Faciale; Université Paris-Saclay, Faculté de Médecine
**Primary Subject Heading**:	Epidemiology
Secondary Subject Heading:	Ear, nose and throat/otolaryngology
Keywords:	Chronic airways disease < THORACIC MEDICINE, Epidemiology < THORACIC MEDICINE, RESPIRATORY MEDICINE (see Thoracic Medicine), Audiology < OTOLARYNGOLOGY, Paediatric otolaryngology < OTOLARYNGOLOGY, Paediatric thoracic medicine < THORACIC MEDICINE

SCHOLARONE™
Manuscripts

I, the Submitting Author has the right to grant and does grant on behalf of all authors of the Work (as defined in the below author licence), an exclusive licence and/or a non-exclusive licence for contributions from authors who are: i) UK Crown employees; ii) where BMJ has agreed a CC-BY licence shall apply, and/or iii) in accordance with the terms applicable for US Federal Government officers or employees acting as part of their official duties; on a worldwide, perpetual, irrevocable, royalty-free basis to BMJ Publishing Group Ltd ("BMJ") its licensees and where the relevant Journal is co-owned by BMJ to the co-owners of the Journal, to publish the Work in this journal and any other BMJ products and to exploit all rights, as set out in our licence.

The Submitting Author accepts and understands that any supply made under these terms is made by BMJ to the Submitting Author unless you are acting as an employee on behalf of your employer or a postgraduate student of an affiliated institution which is paying any applicable article publishing charge ("APC") for Open Access articles. Where the Submitting Author wishes to make the Work available on an Open Access basis (and intends to pay the relevant APC), the terms of reuse of such Open Access shall be governed by a Creative Commons licence – details of these licences and which Creative Commons licence will apply to this Work are set out in our licence referred to above.

Other than as permitted in any relevant BMJ Author's Self Archiving Policies, I confirm this Work has not been accepted for publication elsewhere, is not being considered for publication elsewhere and does not duplicate material already published. I confirm all authors consent to publication of this Work and authorise the granting of this licence.

For peer review only

Title page

Study protocol: the Ear-Nose-Throat (ENT) Prospective International Cohort of patients with Primary Ciliary Dyskinesia (EPIC-PCD)

Myrofora Goutaki[1-2], Yin Ting Lam[1], Mihaela Alexandru[3-4], Andreas Anagiotos[5], Miguel Armengot[6-7], Emilie Bequignon[8], Mieke Boon[9], Andrea Burgess[10], Andre Coste[8,11], Nagehan Emiralioglu[12], Ela Erdem[13], Eric G. Haarman[14], Amanda Harris[15], Sara-Lynn Hool[16], Bulent Karadag[13], Sookyung Kim[3], Philipp Latzin[2], Natalie Lorent[17], Ugur Ozcelik[12], Ana Reula[18-19], Jobst Roehmel[20], Christine van Gogh[21], Panayiotis Yiallouros[22-23], Soeren Marian Zappe[24], for the EPIC-PCD team*, Jean-François Papon[3,25]

Affiliations:

1. Institute of Social and Preventive Medicine, University of Bern, Switzerland.
2. Paediatric Respiratory Medicine, Children's University Hospital of Bern, University of Bern, Switzerland.
3. AP-HP, Hôpital Kremlin-Bicetre, Service d'ORL et de Chirurgie Cervico-Faciale, Le Kremlin-Bicêtre, France.
4. Faculté de Médecine, Université Paris-Saclay, Le Kremlin-Bicêtre, France.
5. Department of Otorhinolaryngology, Nicosia General Hospital, Nicosia, Cyprus.
6. Department of Otorhinolaryngology, and Primary Ciliary Dyskinesia Unit, La Fe University and Polytechnic Hospital, Valencia, Spain.
7. Medical School, Valencia University, Valencia, Spain.
8. AP-HP, Hôpital Henri Mondor et Centre Hospitalier Intercommunal de Créteil, Service d'Oto-Rhino-Laryngologie et de Chirurgie Cervico-Faciale, Créteil, France.
9. Department of Paediatrics, University Hospital, Leuven, Belgium.
10. Primary Ciliary Dyskinesia Centre, Southampton Children's Hospital, Southampton NHS Foundation Trust, Southampton, UK.
11. Faculté de Médecine, Université Paris-Est Créteil, Institut National de la Santé et de la Recherche Médicale, Centre National de la Recherche Scientifique, Hôpital Henri Mondor, Créteil, France.
12. Department of Pediatric Pulmonology, Hacettepe University, School of Medicine, Ankara, Turkey.
13. Department of Pediatric Pulmonology, Marmara University, School of Medicine, Istanbul, Turkey.
14. Department of pediatric pulmonology, Emma Children's Hospital, Amsterdam UMC, Vrije Universiteit, Amsterdam, The Netherlands.
15. Primary Ciliary Dyskinesia Centre, NIHR Respiratory Biomedical Research Centre, University of Southampton, Southampton, UK.

For peer review only - http://bmjopen.bmj.com/site/about/guidelines.xhtml

16. Department of Otorhinolaryngology, Head and Neck Surgery, University Hospital of Bern, University of Bern, Switzerland.
17. Department of Respiratory Diseases, University Hospital, Leuven, Belgium.
18. Biomedical Sciences Department, CEU-Cardenal Herrera University, Castellón, Spain.
19. Molecular, Cellular and Genomic Biomedicine Group, IIS La Fe, Valencia, Spain.
20. Department of Pediatric Pulmonology, Immunology and Critical Care Medicine, Charité-Universitätsmedizin, Berlin, Germany.
21. Department of Otolaryngology/ Head and Neck Surgery, Amsterdam University Medical Center, Amsterdam, The Netherlands.
22. Medical School, University of Cyprus, Nicosia, Cyprus.
23. Pediatric Pulmonology Unit, Hospital 'Archbishop Makarios III', Nicosia, Cyprus.
24. Department of Otorhinolaryngology, Head and Neck Surgery, Charité-Universitätsmedizin, Berlin, Germany.
25. Faculté de Médecine, Université Paris-Saclay, Le Kremlin-Bicêtre, France.

Correspondence: Myrofora Goutaki, Institute of Social and Preventive Medicine, University of Bern, Mittelstrasse 43, 3012 Bern, Switzerland. myrofora.goutaki@ispm.unibe.ch ; Tel.: +41 31 684 5973

*Full list of the EPIC-PCD team collaborators on page 18

For peer review only

Abstract

Introduction: Primary Ciliary Dyskinesia (PCD) is a rare, genetic, multiorgan disease with an estimated prevalence of 1 in 10,000. It affects mainly the upper and lower airways due to impaired mucociliary clearance. Almost all patients have sinonasal or otologic (ear-nose-throat, ENT) problems although the ENT clinical phenotype may present great variability. Despite that, data on PCD ENT manifestations are scarce and based on small single-centre studies. To date we know little about the spectrum and severity of PCD ENT disease, its association with lung disease, its course over life, and its determinants of prognosis.

This study protocol describes the aims and methods of the first prospective, observational, multinational cohort study focusing on ENT disease in PCD patients.

Methods and Analysis: The ENT Prospective International Cohort of PCD patients (EPIC-PCD) is a prospective standardised observational clinical cohort set up as a multinational multi-centre study, embedded into routine patient care. It aims to longitudinally characterise ENT disease in PCD patients and its association with lung disease, and to identify determinants of its prognosis. Patients of all ages, diagnosed with PCD who undergo an ENT clinical assessment at least once a year at one of the participating centres will be invited to participate. Collected data include diagnostic test results, results of ENT examinations, lung function measurements, information on management of ENT disease and patient-reported data on clinical symptoms and health-related quality of life (QoL). Data are collected using the standardised PCD-specific FOLLOW-PCD form and the validated QoL-PCD questionnaire.

Ethics and dissemination: The study has been reviewed and approved by the Human Research Ethics Committees at all participating centres, based on local legislation. The results of the study will be published in scientific journals, presented at scientific conferences and disseminated to participants and national patient organizations.

For peer review only - http://bmjopen.bmj.com/site/about/guidelines.xhtml

The study is registered at ClinicalTrials.gov (NCT04611516).

Keywords: Primary Ciliary Dyskinesia, Orphan disease, Epidemiology, Cohort study, Upper airways, rhinology, otology

For peer review only

Strengths and limitations of this study

- The EPIC-PCD is the first prospective international multicentre clinical study focusing on upper airways disease in patients with PCD.
- The study is embedded in routine patient care, which allows recruitment of large numbers of patients, necessary in rare diseases research.
- The study combines objective assessments of the upper airways and patient reported information on symptoms and health-related quality of life, all collected with disease-specific tools, in a standardised way.
- The cohort aims to be representative of paediatric and adult patients with PCD, followed up at established PCD clinical centres, however it will not represent patients with atypical symptoms who remain undiagnosed or live in areas where diagnosis and care of PCD is not well established.
- The EPIC-PCD has the potential to become a valuable research resource in order to help understand better the disease and to improve diagnosis, clinical care and quality of life of patients with PCD.

For peer review only

Introduction

Primary ciliary dyskinesia (PCD) is a rare genetic, multiorgan disorder affecting approximately 1:10,000 individuals.[1 2] Mutations in disease causing-genes result in defects in ciliary structure or function, leading to a wide range of symptoms from different organ systems.[3-5] The clinical phenotype of the disease is heterogeneous, however reduced airway mucociliary clearance predisposes almost all affected individuals to recurrent upper and lower respiratory infections.[6 7]

Persistent symptoms from the upper airways and ears (ear nose throat: ENT) are a common characteristic of most PCD patients. Ear symptoms commonly present in children with PCD as severe and bilateral otitis media with effusion (OME).[8-11] OME usually resolves spontaneously in otherwise healthy children, but in PCD it seems to persist longer and might need specific management.[8 12-15] In some cases, persistent OME may lead to hearing impairment and cause delays in speech development.[16 17] A newly published study evaluated 47 children in two PCD centres in North America and found that 52% of children with PCD-related hearing loss were not aware of their hearing deficit that was present on audiological assessment.[18] While otologic involvement seems to improve with age in PCD patients, sinonasal disease appears to persist and may worsen in adulthood.[19] First manifestations appear sometimes soon after birth and are usually nonspecific in the form of rhinitis and episodic facial pain due to recurrent infections leading gradually to chronic rhinosinusitis (CRS).[20-24] In adults, a recent study identified sinonasal disease as a primary cause of decreased quality of life in PCD patients.[19] Hypoplasia and agenesis of paranasal sinuses have also been described, while nasal polyps are more common in PCD patients compared to the general population, although this complication has not been well characterised. [19 25 26]

Despite being so frequent and impairing patients' physical health and quality of life (QoL), data on ENT disease in PCD are still scarce. Several large national and multinational research collaborations have made important advances in PCD research in the recent years, though most clinical studies focus on improving diagnosis and characterising lung disease.[27-30] Only few retrospective and single-

For peer review only - http://bmjopen.bmj.com/site/about/guidelines.xhtml

centre studies have described ENT manifestations is some detail. Most studies focused on children and little is known about age-related changes,[8 13] while studies including standardised sinonasal and otologic evaluations were rare. We lack knowledge about the spectrum and severity of ENT disease in patients with PCD, its association with lung disease, its course over life, and determinants of prognosis.

This study protocol describes the aims and methods of the first prospective, observational, multinational cohort study focusing on ENT disease in PCD patients.

Study objectives

This study aims to characterise ENT disease in PCD patients and its association with lung disease, and to identify determinants of its course and prognosis. Specifically, we aim to:

a) Assess the prevalence and severity of sinonasal and otologic symptoms and the frequency and range of signs and physiological findings assessed during standardised ENT physical examination, and describe possible differences by age;
b) Study the association of sinonasal and otologic disease with lung disease in PCD patients;
c) Identify determinants of the course and prognosis of sinonasal and otologic disease in PCD patients.

Methods and analysis

Study design

The ENT Prospective International Cohort of PCD patients (EPIC-PCD) is a prospective observational clinical cohort set up as a multinational multi-centre study. It is embedded into routine patient care of participating reference centres for PCD and patients continue to be managed according to local procedures and protocols. The study is hosted at the Institute of Social and Preventive Medicine (ISPM) (EPIC-PCD data centre) at the University of Bern, Switzerland, and is managed in collaboration with participating centres (EPIC-PCD clinics) (ClinicalTrials.gov Identifier: NCT04611516).

For peer review only - http://bmjopen.bmj.com/site/about/guidelines.xhtml

Patient data are collected prospectively. Baseline information is collected at the enrolment of the patient in the study and follow-up data during clinical follow-up visits at the participating centres. The study has no foreseen endpoint. Patient follow-up is planned for a minimum period of 4 years, however it will be extended if collaborators agree.

Study population

Inclusion criteria and participating centres

Patients of all ages, diagnosed with PCD, according to the European Respiratory Society (ERS) guidelines, and followed-up at one of the participating centres are invited to participate to the EPIC-PCD.[27] Patients are excluded if they do not undergo an ENT clinical assessment at baseline and at least once a year during the study period.

Patient recruitment has started at the following centres (EPIC-PCD clinics; Figure 1):

- University Hospital Bicêtre Paris-Sud, France;
- Centre Hospitalier Intercommunal Creteil and University Hospital Henri Mondor, France;
- Hospital Universitario La Fe in Valencia, Spain;
- University Hospital of Southampton, United Kingdom;
- University Hospital of Leuven, Belgium;
- VU University medical center (VUmc) in Amsterdam, The Netherlands;
- University of Cyprus;
- Hacettepe University in Ankara, Turkey;
- Marmara University in Istanbul, Turkey;
- University Children´s Hospital Charité-Universitätsmedizin in Berlin, Germany, and
- University Children's hospital in Bern Switzerland.

After the establishment of the study in these initial EPIC-PCD clinics, we will invite more PCD centres to join the study and begin patient recruitment.

For peer review only - http://bmjopen.bmj.com/site/about/guidelines.xhtml

Study procedures

Patient identification and recruitment

Eligible patients are identified and invited to participate in the study, and receive all study information by the local clinical team at the EPIC-PCD clinics. Patients are recruited during a clinical follow-up visit; there might be small variations in recruitment and data collection between participating centres to allow the study to be embedded in the regular clinical routine.

Clinical assessments

Eligible patients are followed regularly at the EPIC-PCD clinics, at 3 to 6-month intervals, as planned for clinical reasons, regardless of their participation in the study. Each patient undergoes a detailed sinonasal and otologic examination by ENT specialists, at minimum once a year, during a scheduled follow-up visit. Additional ENT examinations are performed upon clinical indication in-between follow-up visits. Patients will not be subjected to additional invasive measurements solely for the purposes of the study; examinations and tests are only done if clinically indicated or as part of the local follow-up protocol. All examinations and tests are performed following the local Standard Operating Procedures (SOPs) of the clinic. The extended study team discusses differences and ensures that collected data are standardised and comparable.

Data collection instruments

For the collection of patient data, we use standardised PCD-specific instruments, specifically FOLLOW-PCD and QoL-PCD.

FOLLOW-PCD is a disease-specific form for standardised prospective data collection during routine clinical follow-up of PCD patients.[31] It was developed by a multidisciplinary, international working group of the BEAT-PCD COST Action network (Better Experimental Approaches to Treat PCD).[28 32] FOLLOW-PCD has a modular structure, which permits different members of the clinical team (pulmonologists, ENT physicians, diagnostic experts, physiotherapists, lung function technicians etc.) to complete different modules. FOLLOW-PCD consists of seven modules that enable an extensive

For peer review only - http://bmjopen.bmj.com/site/about/guidelines.xhtml

evaluation at baseline and annual reviews, when all modules are completed, and a shorter evaluation at routine (e.g. 3-monthly) follow-up, where only a few modules are used. For the EPIC-PCD study, we use specific modules to collect clinical data relevant to the study, particularly demographic and diagnostic information, results of the physical ENT examination, results of clinical tests and data on hospitalizations and treatments related to the upper airways.

Some modules of FOLLOW-PCD have been developed into patient questionnaires (FOLLOW-PCD questionnaire) that can be completed by the patients during each clinical follow-up visit. The FOLLOW-PCD questionnaire includes detailed questions on frequency and severity of upper and lower respiratory symptoms and health-related behaviours (e.g. smoking, exercise).[31] There are three age-specific versions of the questionnaires: one for adult patients, one for adolescents 14-18 years old and one for the parents or caretakers of patients aged less than 14 years. The FOLLOW-PCD questionnaire has been originally developed in English, German and Greek and is translated in additional languages using a standardised procedure.

In addition to the FOLLOW-PCD questionnaire, we will ask the patients to complete once a year the **QoL-PCD**, a disease-specific, health-related quality of life instrument for PCD, validated and translated in several languages. [33-36] There are four different versions available for a) children (6-12 years), b) adolescents (13-17 years), c) adults (≥18 years), and d) a parent-proxy measure for children ≤12 years. The different versions of the questionnaire are divided into 7-10 hypothesised scales generated by multitrait analysis. All versions include two scales about upper respiratory symptoms, and ears and hearing symptoms, which are particularly relevant to this project. The QoL-PCD has demonstrated good internal consistency and test-retest reliability, and is a valuable tool for observational and interventional studies in PCD.

The FOLLOW-PCD questionnaire, does not address QoL, therefore the two questionnaires provide important complimentary information. Both questionnaires have been translated in the local language of each participating EPIC-PCD clinics.

For peer review only - http://bmjopen.bmj.com/site/about/guidelines.xhtml

Lastly, some centres use as part of their regular patient follow-up the Sino-nasal Outcome Test-22 (SNOT-22) questionnaire that assess sinusitis-related QoL.[37] In these centres, we will also record the SNOT-22 score at baseline and at follow-up for all participating patients.

What information is collected

Figure 2 provides a brief overview of the information collected from participating patients at the baseline and follow-up visits, including the minimum required data, which are assessed for all patients in all participating centres and additional data from tests performed in few centres and which will be collected if available for subgroup analysis to address additional research questions. Small exemptions are allowed when a minimally required examination was not performed due to e.g. the patient's very young age, as long as each patient undergoes a specialised ENT examination at least once a year.

ENT examinations

The ENT examinations performed in the participating centres in addition to physical examination include nasal endoscopy (either rigid or flexible), nasal function tests (e.g. patency, olfaction) tests, tympanometry, audiometry, and auditory brainstem evoked response. Nasal endoscopy, tympanometry and audiometry are performed regularly at all centres during follow-up and belong to the minimum requirements for participation to the study; the remaining tests are performed only in selected, specialised centres either at initial diagnosis or during follow-up. When clinically indicated, patients will undergo computed tomography of the sinuses or the ears. When available, we will collect data of previous imaging examinations of the sinuses or the ears from clinical records.

The data collected during the ENT examinations are predefined based on the ENT examinations module of the FOLLOW-PCD standardised form.[31] Nasal endoscopy data include presence and consistency of nasal discharge (e.g. serous, muco-purulent), evaluation of nasal mucosa (e.g. presence of erythema or oedema), visual evaluation of nasal polyps using the Lildholdt score, [38] evaluation of hypertrophy or atrophy of nasal turbinates and description of septum and any deformities caused due to chronic infection. Otoscopy data include presence and consistency of ear

For peer review only - http://bmjopen.bmj.com/site/about/guidelines.xhtml

discharge, visual description of the tympanic membrane (e.g. tympanic perforation, retracted membrane, tympanic sclerosis) and the presence of acute otitis media or OME. Results of tympanometry are recorded using the description of tympanogram type by Jerger and of audiometry using the type of audiometry and the World Health Organization hearing loss grades.[39] In addition, we record the presence of grommets or use of hearing aids. When sinus imaging is performed, we record the following findings: presence of aplasia, hypoplasia, thickening of the bone and Lund-Mackay score.[40]

Other data from clinical records

At recruitment, baseline medical information and results of diagnostic tests will be extracted from clinical records and recorded using FOLLOW-PCD. In addition to the medical data from the upper airways, we collect data from lung function measurements including spirometry and when available multiple breath-washout measurements and lung imaging. This information will allow us to assess associations between upper and lower respiratory disease in PCD.

Upon indication, nasopharyngeal or nasal swab samples, and when possible middle meatal samples, and sputum or cough samples for microbiological cultures and results will be collected. In case of otorrhea, ear drainage samples will also be taken. We record the type of sample, the isolated pathogens and antibiotic resistance. We also collect information related to hospitalisations and surgical procedures related to upper airways disease and data on upper airway management.

Information from questionnaires

Participating patients are asked to complete the FOLLOW-PCD questionnaire at every visit and the QoL-PCD questionnaire at least once a year, as described earlier. The FOLLOW-PCD questionnaire includes sections on upper and lower respiratory symptoms, health-related behaviours (e.g. exercise, smoking) and environmental factors. The QoL-PCD questionnaire assesses how the disease affects the quality of life of PCD patients.

For peer review only

For peer review only - http://bmjopen.bmj.com/site/about/guidelines.xhtml

Study database

All collected data are entered into a secure web-based database using the database program Research Electronic Data Capture (REDCap) hosted by the Clinical Trials Unit (CTU) Bern.[41] REDCap is widely used in academic research and allows data entry and extraction in various formats. Contributing centres are responsible for the pseudonymisation of the data contributed to the study. Only limited research staff from the EPIC-PCD data team has access to the whole dataset; local investigators at the EPIC-PCD clinics have access only to data from their clinic (text box 1). Data generation, transmission, storage and analysis of health-related personal data within the study follow the Swiss legal requirements for data protection. All study team members are trained on data protection and dealing with particularly sensitive data.

Study power and sample size

We expect to recruit at least 450-550 patients in EPIC-PCD. This number is based on the study feasibility and the number of patients followed-up in the EPIC-PCD clinics and is considered large enough for a prospective study in such a rare disease and much larger than any other available PCD dataset on ENT disease. After the establishment of the cohort, it is now possible for more centres to join and contribute patients, resulting in even higher numbers of patients. The planned analyses are up to a point exploratory and do not follow specific hypotheses, although we have few assumptions based on the limited literature. This does not allow for a precise power calculation; however we will ensure that each performed analysis has sufficient power to draw meaningful conclusions.

Text Box 1: Contributing to and accessing data in the ENT Prospective International Cohort of PCD patients (EPIC-PCD)

How to contribute data

- Centres that wish to participate in the project and contribute data can contact the EPIC-PCD data team. As soon as ethical approval is obtained and centre representatives have signed a data delivery agreement, they will receive a password to access the online software REDCap and they

For peer review only

will be able to enter their data directly prospectively. We offer information and technical support wherever needed.

How to access data

- Participating centres keep constant access to their datasets and can export them directly in various formats for local analyses. Participating centres may also extract their datasets and contribute relevant data to other multinational projects or to the international PCD registry.
- The use of EPIC-PCD data from several centres for studies and publications is regulated by the EPIC-PCD committee, which consists of members of the EPIC-PCD data centre and representatives of the EPIC-PCD clinics. A series of publications are originally planned to be led by the EPIC-PCD data team, as part of the research grant funding the set-up and management of the study.
- For additional publications, collaborators should submit a concept sheet describing the planned analysis and publication to the committee, which will approve or oppose the planned publication and use of data. If the planned analysis is approved, a publication agreement will be signed and the EPIC-PCD data centre will prepare a partial dataset for the proposed analysis and will work closely with the lead researchers of each planned study for methodological input and support.
- For further details, contact: myrofora.goutaki@ispm.unibe.ch.

REDCap: Research Electronic Data Capture.

For peer review only

Planned analysis and outcomes of interest

First, we will describe prevalence and severity of ENT problems, stratified by age, age at diagnosis, sex, country, ciliary ultrastructural defect or genetic mutation. Outcomes of interest will be sinonasal and otologic symptoms and signs (e.g. rhinitis, loss of smell, sinusitis, otitis, impaired hearing) and the results of ENT examinations (mainly of nasal endoscopy, tympanometry, audiometry and when available imaging and SNOT-22). We will estimate prevalence of binary outcomes and mean values of continuous outcomes by using constant-only models. We will use logistic regression models for

For peer review only - http://bmjopen.bmj.com/site/about/guidelines.xhtml

binary outcomes, and linear models for continuous outcomes. Associations with determinants will be assessed in multivariable mixed models.

As a second step, we will assess the association of baseline ENT problems with lung disease progression expressed in terms of lung function tests, sputum cultures and, if available, lung imaging results. For this analysis, lung function will be the main outcome, measured primarily with spirometry and plethysmography measuring forced expiratory vital capacity (FVC) and forced expiratory volume in 1 second (FEV1). Data will be converted into z-scores adjusted for sex, height, age, and ethnicity, using the Global Lung Initiative (GLI) reference values. In some centres, PCD patients also perform multiple breath washout measurements at follow-up, which have been reported to be more sensitive in detecting early lung disease. In those patients, lung clearance index (LCI) will be considered as a secondary outcome, if patients´ numbers allow it. We will use mixed models, including the baseline ENT disease characteristics with an interaction of each with age as fixed effects and the patients and the centres as random effects.

Finally, we will analyse follow-up data on ENT symptoms and examinations to describe the short-term course of sinonasal and otologic disease during a 2-year follow-up, and identify determinants of disease prognosis. Here, outcomes of interest will be the severity of CRS (based on a symptoms score), loss of smell, bacterial colonization of the upper respiratory tract, need of surgical management of the sinuses, recurrent otitis media, OME, hearing impairment, and need of surgical management of the ears. We will first describe how the outcomes change over the follow-up time in binomial or multinomial (for outcomes with more than three categories such as hearing impairment) mixed models, using separate models for each outcome. Then, we will study determinants of disease prognosis categorising patients in four groups for each outcome based on the evaluation at baseline and at 2-year follow-up: a) patients who never reported the outcome, b) patients negative for the outcome at baseline but positive at follow-up, c) patients positive at baseline but negative at follow-up and d) patients positive at both time points. We will use mixed-effects multinomial regression models, including all possible determinants as fixed effects. We will select possible determinants

For peer review only - http://bmjopen.bmj.com/site/about/guidelines.xhtml

from characteristics available at baseline including symptoms and measurements; age at diagnosis, ultrastructural defects, and genetic mutations; and general patient characteristics such as age and sex. We will make our determinants selection based on directed acyclic graphs, considering existing literature. Data will be collected prospectively and in a standardised way resulting in small data heterogeneity between participating centres, however we will still need to account for possible differences between countries in all analyses. Thus, we will analyse all outcomes of interest using random-effects models that account for clustering of patients within participating centres.

For all analyses, results will be interpreted with caution considering the clinical importance and not just mere statistically significant findings. If needed, we will correct for multiple testing. All missing values will be described in detail, however we expect this will not be an issue for the important variables as the data is collected prospectively. In case we have significant amount of missing data, we will consider subgroup analyses or multiple imputation of missing data to deal with this issue, depending on what is the most appropriate approach for the specific question and analysis.

Ethics and dissemination

Ethical approval to share pseudo-anonymised patient data was obtained locally at each participating centre (Supplementary file). Written informed consent will be obtained in accordance with the national/local data protection laws, from patients aged 14 years or more (might differ per country based on local laws) or from the patient's parents/caregivers for younger patients. Patients can withdraw their consent and their data from the study at any time by contacting the clinical team at each participating centre.

The results of the study will be published in scientific journals and presented at scientific conferences to raise awareness about upper respiratory disease in PCD patients.

The principal investigator of the study affirms and upholds the principle of the participants' right to dignity, privacy and health and that the project team shall comply with applicable privacy laws. Especially, anonymity of the participants shall be guaranteed when presenting the data at scientific meetings or publishing them in scientific journals. Individual participant medical information

For peer review only - http://bmjopen.bmj.com/site/about/guidelines.xhtml

obtained during this research project is considered confidential and will not be disclosed to third parties. Data will not be deposited at an open access repository. Because of the rarity of the disease although data will be pseudonymised, it may include still sensitive information, therefore participants will not be asked to give consent to have their data deposited publicly.

Patient and public involvement

Several patient support organizations were consulted and support the study. The EPIC-PCD is set up and can be further developed in the framework of the BEAT-PCD clinical research collaboration (CRC) network supported by the European Respiratory Society[42]. The BEAT-PCD CRC is a large multidisciplinary network, which includes more than 500 researchers and clinicians from more than 30 countries. The EPIC-PCD is part of the BEAT-PCD research portfolio, which ensures widespread dissemination of the study results to researchers, clinicians, patients and all relevant stakeholders. Additionally, the EPIC-PCD study team works closely with a team of PCD patients who consult BEAT-PCD to ensure that all studies support their interests. Summaries of results in lay language will be disseminated to participants and national patient organizations by the EPIC-PCD data centre and the EPIC-PCD clinics and to the relevant stakeholders and the public through the BEAT-PCD CRC website and social media.

For peer review only

For peer review only - http://bmjopen.bmj.com/site/about/guidelines.xhtml

***For the EPIC-PCD team** (listed in alphabetical order):
Dilber Ademhan (Hacettepe University, Turkey), Mihaela Alexandru (AP-HP, France), Andreas Anagiotos (Nicosia General Hospital, Cyprus), Miguel Armengot (La Fe University and Polytechnic Hospital, Spain), Emilie Bequignon (AP-HP, France), Irma Bon (Vrije Universiteit, The Netherlands), Mieke Boon (University Hospital Leuven, Belgium), Marina Bullo (University of Bern, Switzerland), Andrea Burgess (University of Southampton, UK), Carmen Casaulta (University of Bern, Switzerland), Marco Caversaccio (University of Bern, Switzerland), Nathalie Caversaccio (University of Bern, Switzerland), Andre Coste (AP-HP, France), Bruno Crestani (RESPIRARE, France), Sandra Diepenhorst (Vrije Universiteit, The Netherlands), Nagehan Emiralioglu (Hacettepe University, Turkey), Ela Erdem (Marmara University, Turkey), Pinar Ergenekon (Marmara University, Turkey), Nathalie Feyaerts (University Hospital Leuven, Belgium), Gabriel Georgiou (Nicosia General Hospital, Cyprus), Amy Glen (University of Southampton, UK), Christine van Gogh (Vrije Universiteit, The Netherlands), Yasemin Gokdemir (Marmara University, Turkey), Myrofora Goutaki (University of Bern, Switzerland), Onder Gunaydın (Hacettepe University, Turkey), Eric G. Haarman (Vrije Universiteit, The Netherlands), Amanda Harris (University of Southampton, UK), Sara-Lynn Hool (University of Bern, Switzerland), Isabelle Honoré (RESPIRARE, France), Hasnaa Ismail-Koch (University of Southampton, UK), Bulent Karadag (Marmara University, Turkey), Elisabeth Kieninger (University of Bern, Switzerland), Sookyung Kim (AP-HP, France), Panayiotis Kouis (University of Cyprus, Cyprus), Bernard Maitre (RESPIRARE, France), David Montani (RESPIRARE, France), Yin Ting Lam (University of Bern, Switzerland), Philipp Latzin (University of Bern, Switzerland), Marie Legendre (RESPIRARE, France), Natalie Lorent (University Hospital Leuven, Belgium), Jane S. Lucas (University of Southampton, UK), Alison McEvoy (University of Southampton, UK), Rana Mitri-Frangieh (RESPIRARE, France), Loretta Müller (University of Bern, Switzerland), Noelia Muñoz (La Fe University and Polytechnic Hospital, Spain), Ugur Ozcelik (Hacettepe University, Turkey), Beste Ozsezen (Hacettepe University, Turkey), Samantha Packham (University of Southampton, UK), Jean-François Papon (AP-HP, France), Ana Reula (La Fe University, Spain), Jobst Roehmel (Charité-Universitätsmedizin Berlin, Germany), Simone Tanner (Vrije Universiteit, The Netherlands), Guillaume Thouvenin (RESPIRARE, France), Woolf T. Walker (University of Southampton, UK), Hannah Wilkins (University of Southampton, UK), Panayiotis Yiallouros (University of Cyprus, Cyprus), Soeren Marian Zappe (Charité-Universitätsmedizin Berlin, Germany).

For peer review only - http://bmjopen.bmj.com/site/about/guidelines.xhtml

Authors' contributions: MG conceptualized and designed the study. MG, YTL and JFP drafted the protocol. YTL, MA, AA, MAr, EB, MB, AB, AC, NE, EE, EGH, AH, SLH, BK, SK, PL, NL, UO, AR, JR, CvG, PY, SMZ and JFP are responsible for the study conduct and data acquisition. MG and YTL are responsible for data analysis. MG, YTL, MA, AA, MAr, EB, MB, AB, AC, NE, EE, EGH, AH, SLH, BK, SK, PL, NL, UO, AR, JR, CvG, PY, SMZ and JFP are responsible for the data interpretation and have critically revised and approved this manuscript.

Funding statement: This work was supported by the Swiss National Science Foundation grant number (SNFPZ00P3_185923). Future grant applications by all partners to different funding bodies will provide continuous support. Authors participate in the BEAT-PCD clinical research collaboration, supported by the European Respiratory Society and most participating centres are part of the ERN-LUNG (European Reference Network) PCD-Core.

Competing interests statement: The authors have no conflict of interest related to this study. Dr. Latzin reports personal fees from Gilead, Novartis, OM pharma, Polyphor, Roche, Santhera, Schwabe, Vertex, Vifor, Zambon and grants from Vertex, all outside the submitted work.

Acknowledgements: We thank all the patients with PCD in the cohort and their families, and the PCD support organisations (especially: PCD Family Support Group UK; Association ADCP France; Kartagener Syndrom und Primäre Ciliäre Dyskinesie e. V. Deutschland/ Deutschschweiz) for their close collaboration. We also thank all the researchers in the participating centres who are involved in recruitment, data collection and data entry, and work closely with us through the whole process of participating to the EPIC-PCD.

For peer review only

References

1. Kuehni CE, Frischer T, Strippoli MP, et al. Factors influencing age at diagnosis of primary ciliary dyskinesia in European children. *The European respiratory journal* 2010;36(6):1248-58. doi: 10.1183/09031936.00001010 [published Online First: 2010/06/10]
2. Lucas JS, Walker WT, Kuehni CE, et al. Primary Ciliary Dyskinesia. *In: Courdier J-F, editor Orphan Lung diseases European Respiratory Monograph* 2011:201-17.
3. Kennedy MP, Omran H, Leigh MW, et al. Congenital heart disease and other heterotaxic defects in a large cohort of patients with primary ciliary dyskinesia. *Circulation* 2007;115(22):2814-21. doi: 10.1161/CIRCULATIONAHA.106.649038 [published Online First: 2007/05/23]
4. Shapiro AJ, Davis SD, Ferkol T, et al. Laterality defects other than situs inversus totalis in primary ciliary dyskinesia: insights into situs ambiguus and heterotaxy. *Chest* 2014;146(5):1176-86. doi: 10.1378/chest.13-1704 [published Online First: 2014/03/01]
5. Vanaken GJ, Bassinet L, Boon M, et al. Infertility in an adult cohort with primary ciliary dyskinesia: phenotype-gene association. *The European respiratory journal* 2017;50(5) doi: 10.1183/13993003.00314-2017 [published Online First: 2017/11/11]
6. Goutaki M, Meier AB, Halbeisen FS, et al. Clinical manifestations in primary ciliary dyskinesia: systematic review and meta-analysis. *The European respiratory journal* 2016;48(4):1081-95. doi: 10.1183/13993003.00736-2016 [published Online First: 2016/08/06]
7. Stannard W, O'Callaghan C. Ciliary function and the role of cilia in clearance. *Journal of aerosol medicine : the official journal of the International Society for Aerosols in Medicine* 2006;19(1):110-5. doi: 10.1089/jam.2006.19.110 [published Online First: 2006/03/23]
8. Pruliere-Escabasse V, Coste A, Chauvin P, et al. Otologic features in children with primary ciliary dyskinesia. *Archives of otolaryngology--head & neck surgery* 2010;136(11):1121-6. doi: 10.1001/archoto.2010.183 [published Online First: 2010/11/17]
9. Sommer JU, Schafer K, Omran H, et al. ENT manifestations in patients with primary ciliary dyskinesia: prevalence and significance of otorhinolaryngologic co-morbidities. *European archives of oto-rhino-laryngology : official journal of the European Federation of Oto-Rhino-Laryngological Societies (EUFOS) : affiliated with the German Society for Oto-Rhino-Laryngology - Head and Neck Surgery* 2011;268(3):383-8. doi: 10.1007/s00405-010-1341-9 [published Online First: 2010/07/24]
10. el-Sayed Y, al-Sarhani A, al-Essa AR. Otological manifestations of primary ciliary dyskinesia. *Clinical otolaryngology and allied sciences* 1997;22(3):266-70. [published Online First: 1997/06/01]
11. Takeuchi K, Kitano M, Sakaida H, et al. Analysis of Otologic Features of Patients With Primary Ciliary Dyskinesia. *Otology & neurotology : official publication of the American Otological Society, American Neurotology Society [and] European*

For peer review only - http://bmjopen.bmj.com/site/about/guidelines.xhtml

Academy of Otology and Neurotology 2017;38(10):e451-e56. doi: 10.1097/MAO.0000000000001599 [published Online First: 2017/11/15]
12. Berman S. Otitis media in children. *The New England journal of medicine* 1995;332(23):1560-5. doi: 10.1056/NEJM199506083322307 [published Online First: 1995/06/08]
13. Majithia A, Fong J, Hariri M, et al. Hearing outcomes in children with primary ciliary dyskinesia—a longitudinal study. *International journal of pediatric otorhinolaryngology* 2005;69(8):1061-64.
14. Campbell RG, Birman CS, Morgan L. Management of otitis media with effusion in children with primary ciliary dyskinesia: a literature review. *International journal of pediatric otorhinolaryngology* 2009;73(12):1630-8. doi: 10.1016/j.ijporl.2009.08.024 [published Online First: 2009/10/03]
15. Majithia A, Fong J, Hariri M, et al. Hearing outcomes in children with primary ciliary dyskinesia--a longitudinal study. *International journal of pediatric otorhinolaryngology* 2005;69(8):1061-4. doi: 10.1016/j.ijporl.2005.02.013 [published Online First: 2005/07/12]
16. Kreicher KL, Schopper HK, Naik AN, et al. Hearing loss in children with primary ciliary dyskinesia. *International journal of pediatric otorhinolaryngology* 2018;104:161-65. doi: 10.1016/j.ijporl.2017.11.005 [published Online First: 2017/12/31]
17. Yoshinaga-Itano C, Sedey AL, Coulter DK, et al. Language of early- and later-identified children with hearing loss. *Pediatrics* 1998;102(5):1161-71. [published Online First: 1998/10/31]
18. Zawawi F, Shapiro AJ, Dell S, et al. Otolaryngology Manifestations of Primary Ciliary Dyskinesia: A Multicenter Study. *Otolaryngol Head Neck Surg* 2021:1945998211019320. doi: 10.1177/01945998211019320 [published Online First: 2021/06/23]
19. Bequignon E, Dupuy L, Zerah-Lancner F, et al. Critical Evaluation of Sinonasal Disease in 64 Adults with Primary Ciliary Dyskinesia. *Journal of clinical medicine* 2019;8(5) doi: 10.3390/jcm8050619 [published Online First: 2019/05/10]
20. Hosie PH, Fitzgerald DA, Jaffe A, et al. Presentation of primary ciliary dyskinesia in children: 30 years' experience. *Journal of paediatrics and child health* 2015;51(7):722-6. doi: 10.1111/jpc.12791 [published Online First: 2014/12/17]
21. Morgan LC, Birman CS. The impact of Primary Ciliary Dyskinesia on the upper respiratory tract. *Paediatric respiratory reviews* 2016;18:33-8. doi: 10.1016/j.prrv.2015.09.006 [published Online First: 2016/02/24]
22. Noone PG, Leigh MW, Sannuti A, et al. Primary ciliary dyskinesia: diagnostic and phenotypic features. *Am J Respir Crit Care Med* 2004;169(4):459-67. doi: 10.1164/rccm.200303-365OC [published Online First: 2003/12/06]
23. Pifferi M, Bush A, Rizzo M, et al. Olfactory dysfunction is worse in primary ciliary dyskinesia compared with other causes of chronic sinusitis in children. *Thorax* 2018;73(10):980-82. doi: 10.1136/thoraxjnl-2017-210661 [published Online First: 2018/03/02]
24. Behan L, Dimitrov BD, Kuehni CE, et al. PICADAR: a diagnostic predictive tool for primary ciliary dyskinesia. *The European respiratory journal* 2016;47(4):1103-12. doi: 10.1183/13993003.01551-2015 [published Online First: 2016/02/27]
25. Campbell R. Managing upper respiratory tract complications of primary ciliary dyskinesia in children. *Current opinion in allergy and clinical immunology* 2012;12(1):32-8. doi: 10.1097/ACI.0b013e32834eccc6 [published Online First: 2011/12/14]
26. Pifferi M, Bush A, Caramella D, et al. Agenesis of paranasal sinuses and nasal nitric oxide in primary ciliary dyskinesia. *The European respiratory journal* 2011;37(3):566-71. doi: 10.1183/09031936.00068810 [published Online First: 2010/07/24]
27. Lucas JS, Barbato A, Collins SA, et al. European Respiratory Society guidelines for the diagnosis of primary ciliary dyskinesia. *The European respiratory journal* 2017;49(1) doi: 10.1183/13993003.01090-2016 [published Online First: 2016/11/12]
28. Rubbo B, Behan L, Dehlink E, et al. Proceedings of the COST action BM1407 inaugural conference BEAT-PCD: translational research in primary ciliary dyskinesia - bench,

For peer review only - http://bmjopen.bmj.com/site/about/guidelines.xhtml

bedside, and population perspectives. *BMC proceedings* 2016;10(Suppl 9):66. doi: 10.1186/s12919-016-0067-0 [published Online First: 2017/08/17]

29. Goutaki M, Maurer E, Halbeisen FS, et al. The international primary ciliary dyskinesia cohort (iPCD Cohort): methods and first results. *The European respiratory journal* 2017;49(1) doi: 10.1183/13993003.01181-2016 [published Online First: 2017/01/06]
30. Halbeisen FS, Goutaki M, Spycher BD, et al. Lung function in patients with primary ciliary dyskinesia: an iPCD Cohort study. *The European respiratory journal* 2018;52(2) doi: 10.1183/13993003.01040-2018 [published Online First: 2018/07/28]
31. Goutaki M, Papon JF, Boon M, et al. Standardised clinical data from patients with primary ciliary dyskinesia (PCD): FOLLOW-PCD *ERJ Open Res Submitted* 2019
32. Gardner LE, Horton KL, Shoemark A, et al. Proceedings of the 4(th) BEAT-PCD Conference and 5(th) PCD Training School. *BMC proceedings* 2020;14(Suppl 8):7. doi: 10.1186/s12919-020-00191-3 [published Online First: 2020/06/25]
33. Dell SD, Leigh MW, Lucas JS, et al. Primary Ciliary Dyskinesia: First Health-related Quality-of-Life Measures for Pediatric Patients. *Annals of the American Thoracic Society* 2016;13(10):1726-35. doi: 10.1513/AnnalsATS.201603-198OC [published Online First: 2016/07/28]
34. Lucas JS, Behan L, Dunn Galvin A, et al. A quality-of-life measure for adults with primary ciliary dyskinesia: QOL-PCD. *The European respiratory journal* 2015;46(2):375-83. doi: 10.1183/09031936.00216214 [published Online First: 2015/05/16]
35. Behan L, Leigh MW, Dell SD, et al. Validation of a health-related quality of life instrument for primary ciliary dyskinesia (QOL-PCD). *Thorax* 2017;72(9):832-39. doi: 10.1136/thoraxjnl-2016-209356 [published Online First: 2017/03/02]
36. Behan L, Leigh MW, Dell SD, et al. Validation of paediatric health-related quality of life instruments for primary ciliary dyskinesia (QOL-PCD). *Pediatric Pulmonology (In Press)* 2019
37. Kennedy J, Hubbard M, Huyett P, et al. Sino-nasal outcome test (SNOT-22): a predictor of postsurgical improvement in patients with chronic sinusitis. *Ann Allergy Asthma Immunol* 2013;111(4):246-251.e2 doi: doi:10.1016/j.anai.2013.06.033
38. Johansson L, Akerlund A, Holmberg K, et al. Evaluation of methods for endoscopic staging of nasal polyposis. *Acta oto-laryngologica* 2000;120(1):72-6. doi: 10.1080/000164800760370873 [published Online First: 2000/04/25]
39. World Health Organization. Report of the informal working group on prevention of deafness and hearing impairment programme planning Geneva1991 [Available from: http://www.who.int/iris/handle/10665/58839 accessed 20.08.2021.
40. Lund VJ, Mackay IS. Staging in rhinosinusitus. *Rhinology* 1993;31(4):183-4. [published Online First: 1993/12/01]
41. Harris PA, Taylor R, Thielke R, et al. Research electronic data capture (REDCap)--a metadata-driven methodology and workflow process for providing translational research informatics support. *Journal of biomedical informatics* 2009;42(2):377-81. doi: 10.1016/j.jbi.2008.08.010 [published Online First: 2008/10/22]
42. Goutaki M, Crowley S, Dehlink E, et al. The BEAT-PCD (Better Experimental Approaches to Treat Primary Ciliary Dyskinesia) Clinical Research Collaboration. *The European respiratory journal* 2021;57(2) doi: 10.1183/13993003.04601-2020 [published Online First: 2021/02/06]

Figure Legends

For peer review only - http://bmjopen.bmj.com/site/about/guidelines.xhtml

Figure 1: Centres currently participating to the EPIC-PCD cohort study.

The EPIC-PCD clinics are marked with a black dot and the EPIC-PCD data centre with a yellow dot.

Figure 2: Overview of the information collected during the EPIC-PCD cohort study

For peer review only

For peer review only - http://bmjopen.bmj.com/site/about/guidelines.xhtml

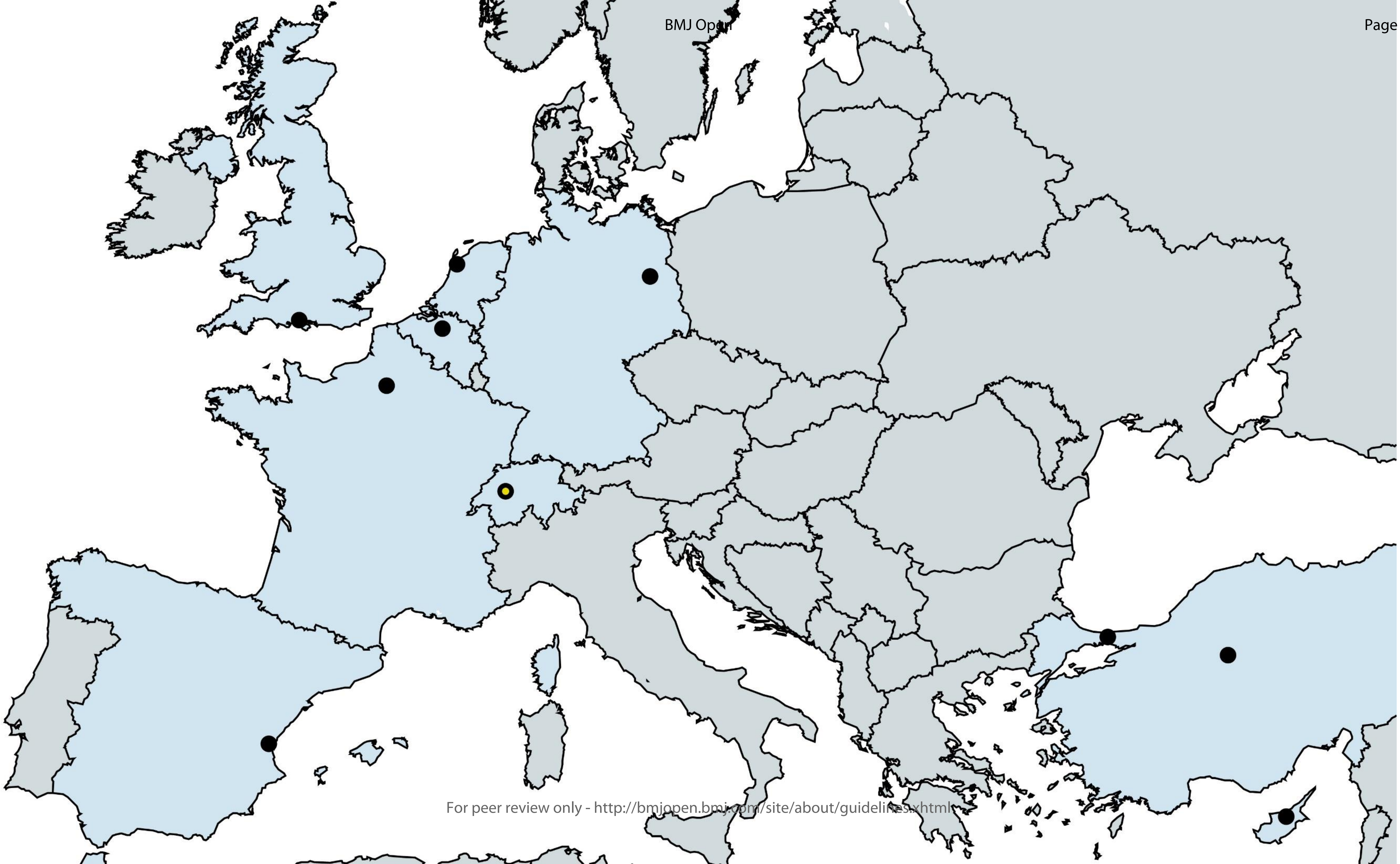

For peer review only - http://bmjopen.bmj.com/site/about/guidelines.xhtml

	BASELINE visit (patient recruitment)	FOLLOW-UP visits (at least once/year)
MINIMUM REQUIREMENTS	• FOLLOW-PCD patient questionnaire<br>• Standardised ENT examination including:<br>Audiometry<br>Tympanometry<br>Nasal endoscopy<br>• Spirometry<br>• Height/Weight<br>• Information on ENT management	• FOLLOW-PCD patient questionnaire<br>• Standardised ENT examination including:<br>Audiometry<br>Tympanometry<br>Nasal endoscopy<br>• Spirometry<br>• Height/Weight<br>• Information on ENT management
	• Diagnostic information<br>• Baseline medical history	
IF AVAILABLE	• QoL-PCD questionnaire/ SNOT-22<br>• Additional ENT examination results e.g.:<br>Nasal function tests/ Audiovestibular function tests/ Auditory brainstem evoked response/ Sinus, ear CT<br>• Plethysmography, Multiple breath washout<br>• Microbiology test results from upper and lower airways	• QoL-PCD questionnaire/ SNOT-22<br>• Additional ENT examination results e.g.:<br>Nasal function tests/ Audiovestibular function tests/ Auditory brainstem evoked response / Sinus, ear CT<br>• Plethysmography, Multiple breath washout<br>• Microbiology test results from upper and lower airways

Supplementary file: Ethics

The study has been reviewed and approved by the local Human Research Ethics Committees at every participating centre, based on local legislation. We list below the names of the ethics committees which approved the study and the approval reference numbers, when applicable.

A. The following centres have a pre-existing or new **ethical approval, which allows the contribution of pseudonymised data to observational collaborative international studies (covers the EPIC-PCD study)**:

- University Children´s Hospital Charité-Universitätsmedizin, **Berlin, Germany:** Ethical Committee Charité (EA2/003/21)
- University Children's hospital, **Bern, Switzerland**: Cantonal Ethics Committee of Bern (KEK-BE: 060/2015)
- University of **Cyprus:** Ethical Committee for biomedical research in Leukosia Cyprus (EEBK/EΠ/2013/21)
- Marmara University **Istanbul, Turkey:** Ethical Committee of Marmara University (09.2018.395)
- University Hospital of **Southampton, United Kingdom:** Southampton and South West Hampshire research ethics committee (06/Q1702/109)

B. The following centres applied for **ethical approval to participate specifically to the EPIC-PCD study**:

- VU University medical center (VUmc), **Amsterdam, The Netherlands:** The Medical Ethics Review Committee of VU University Medical Center reviewed the application and concluded on 24th of November 2020 that no approval is needed to participate to the EPIC-PCD cohort as the Medical Research Involving Human Subjects Act does not apply to the study.
- Hacettepe University, **Ankara, Turkey:** Non-interventional clinical research EC of Hacettepe University (2020/11-47)
- University Hospital of **Leuven, Belgium**: Ethical Committee for Research of University Hospitals Leuven (S64411)
- Hospital Universitario La Fe in **Valencia, Spain:** Ethical Committee of medical investigations of Hospital Universitario La Fe (2020-498-1)
- University Hospital Bicetre Paris-Sud and Centre Hospitalier Intercommuncal Creteil**, Paris, France:** Both hospitals belong to the Assistance Publique–Hôpitaux de Paris and applied for joint approval. The AP-HP Direction de la Recherche Clinique et de l'Innovation reviewed the application and concluded on 4th of February 2021 that that no approval is needed to participate to the EPIC-PCD cohort as the Jardé law that regulates clinical research in France does not apply to the study.

For peer review only
